# Design, Synthesis and Fungicidal Activities of Some Novel Pyrazole Derivatives

**DOI:** 10.3390/molecules190914036

**Published:** 2014-09-08

**Authors:** Xue-Ru Liu, Hua Wu, Ze-Yu He, Zhi-Qing Ma, Jun-Tao Feng, Xing Zhang

**Affiliations:** Research & Development Center of Biorational Pesticide, Key Laboratory of Plant Protection Resources and Pest Management of Ministry of Education, Northwest A&F University, Xinong Road 22, Yangling 712100, Shaanxi, China; E-Mails: xueruliu@nwsuaf.edu.cn (X.-R.L.); wgf20102010@nwsuaf.edu.cn (H.W.); skyhugh2012@gmail.com (Z.-Y.H.); zhiqingma@nwsuaf.edu.cn (Z.-Q.M.); zhxing1952@126.com (X.Z.)

**Keywords:** pyrazole derivatives, chemical modification, antifungal activity, structure–activity relationships

## Abstract

In order to discover new compounds with good fungicidal activities, 32 pyrazole derivatives were designed and synthesized. The structures of the target compounds were confirmed by ^1^H-NMR, ^13^C-NMR, and high-resolution electrospray ionization mass spectrometry (HR-ESI-MS), and their fungicidal activities against *Botrytis cinerea*, *Rhizoctonia solani* Kuhn, *Valsa mali* Miyabe et Yamada, *Thanatephorus cucumeris* (Frank) Donk*, Fusarium oxysporum* (S-chl) f.sp*. cucumerinum* Owen*,* and *Fusarium graminearum* Schw were tested. The bioassay results indicated that most of the derivatives exhibited considerable antifungal activities, especially compound **26** containing a *p*-trifluoromethyl- phenyl moiety showed the highest activity, with EC_50_ values of 2.432, 2.182, 1.787, 1.638, 6.986, and 6.043 μg/mL against *B. cinerea*, *R. solani*, *V. mali*, *T. cucumeris*, *F. oxysporum*, and *F. graminearum*, respectively. Moreover, the activities of compounds such as compounds **27**–**32** were enhanced by introducing isothiocyanate and carboxamide moieties to the 5-position of the pyrazole ring.

## 1. Introduction

It was well accepted that agricultural diseases caused by pathogenic fungi threaten the security and efficiency of crop production [1,2,3]. In order to maintain a substantial increase in crop production and to meet humans’ growing demand for food quality and quantity, fungicides are widely used for crop protection [4]. Unfortunately, many commercial chemical fungicides have been challenged by the development of resistance [5,6]. Thus, there is an urgent need for novel highly active fungicides, especially those with new modes of action, to handle these problems.

In recent years, substituted pyrazole derivatives have captured considerable attention owing to their broad spectrum biological properties. Compounds with pyrazole functional units exhibit antimicrobial [7], herbicidal [8], antitumor [9], insecticidal [10,11,12,13], fungicidal [14,15,16,17,18] and antiviral activities [19,20]. Lamberth [21] has summarized the significance of pyrazole derivatives in crop protection chemistry, including herbicidally-, fungicidally- and insecticidally-active pyrazole classes. The pyrazole ring is a particularly efficient pharmacophore in fungicide design [22]. There are some novel fungicides such as bixafen (Bayer), fluxapyroxad (BASF), penflufen (Bayer), penthiopyrad (Mitsui Chemicals Inc & Co; Dupont), sedaxane (Syngenta), furamethpyr (BASF) and isopyrazam (Syngenta), a group of succinate dehydrogenase inhibitors, which structures include pyrazole rings. These commercial compounds also possess a carboxylic function in position 4. Isothiocyanate compounds such as allyl isothiocyanate, ethyl isothiocyanate, and so on are also used to control fungi mycelial growth [23]. The isothiocyanate group could be combined with zymoprotein which produces sulfydryl amino acids (activation of apoptosis proteins) in the fungi, causing the fungi to die. According to the isostere concept, we have now combined isothiocyanates and substituted pyrazoles to produce a series of novel substituted pyrazole aminopropyl isothiocyanates, seeking compounds with high efficacy fungicidal activity. In addition, the strobilurin derivatives containing both a β-methoxyacrylate and a substituted pyrazole in the side chain displayed excellent fungicidal and acaricidal activities [24]. It was reported that the variation of the substituent and the position on the pyrazole could greatly influence the activities [25,26,27,28,29,30,31,32]. Based on the hypothesis that incorporating different moieties possessing fungicidal properties into the backbone of a pyrazole ring might enhance the fungicidal activities of pyrazole analogues, 32 novel substituted pyrazole derivatives were designed and synthesized in this paper, and the fungicidal activities of these compounds were tested. The structure-activity relationships were also examined.

## 2. Result and Discussion

### 2.1. Chemical Synthesis

**Scheme 1:**
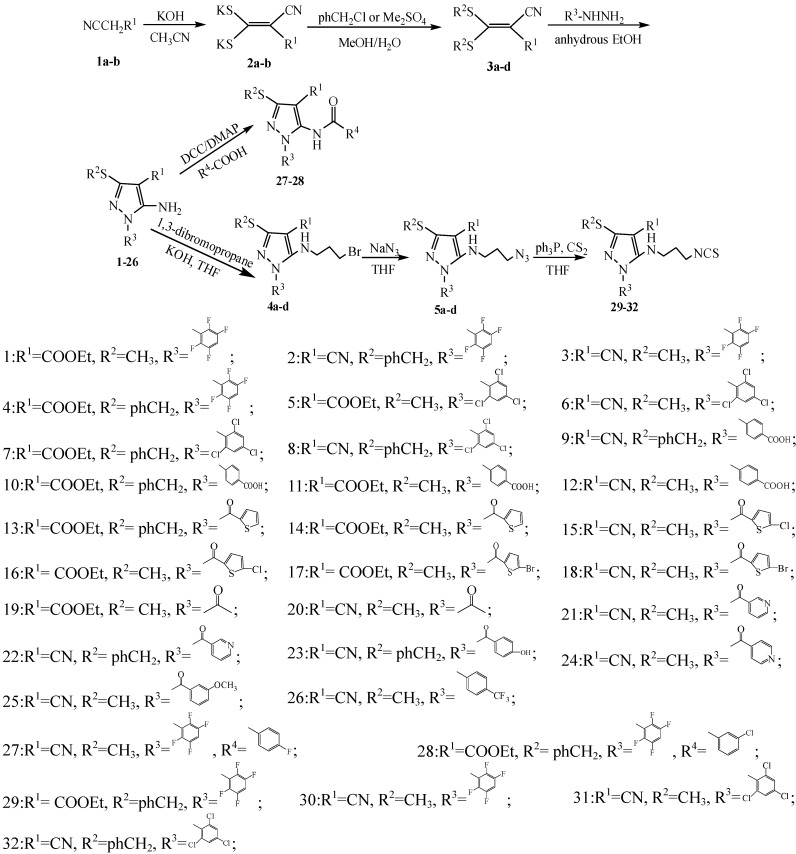
Synthetic route and chemical structures of compounds **1**–**32**.

Thirty-two title compounds were prepared according to the methods presented in Scheme 1, including twenty-six 1,3,4-substituted-5-aminopyrazole derivatives, two 1,3,4-substituted pyrazole amides, and four 1,3,4-substituted pyrazole isothiocyanates. Compounds **3a**–**d** were obtained by the treatment of the dithioacetal dipotassium salts **2a**–**b** with dimethyl sulfate or benzyl chloride in the presence of methanol and H_2_O. The resulting compounds were then reacted with corresponding hydrazines to afford 1,3,4-substituted-5-aminopyrazole derivatives **1**–**26**.

In order to explore the effects of substituted pyrazole compounds against pathogenic fungi, amide groups and isothiocyanate groups were introduced. The substituted pyrazole amides **27** and **28** were synthesized by treating aromatic acid with compounds **3** and **4** in the presence of dicyclohexylcarbodiimide (DCC) and dimethylaminopyridine (DMAP). Preparation of the target 1,3,4-substituted pyrazole isothiocyanate compounds was performed as follows: first, the compounds were converted into 1,3,4-substituted-5-*N*-(3-bromopropyl)pyrazoles through alkylation using 1,3-dibromopropane. Then, **4a**–**d** were reacted with sodium azide to obtain hydrazoate compounds **5a**–**d**. Treatment of **5a**–**d** with triphenylphosphine and carbon disulfide in THF afforded **29**–**32**.

Most of target compounds were synthesized in good yields of 90% or higher and were characterized by ^1^H-NMR, ^13^C-NMR and HR-MS (ESI). All spectral and analytical data were consistent with the assigned structures. 

### 2.2. Fungicidal Activity

The initial screening data results for the fungicidal activities of the tested compounds against *Botrytis cinerea*, *Rhizoctonia solani* Kuhn, *Valsa mali* Miyabe et Yamada, *Thanatephorus cucumeris* (Frank) Donk, *Fusarium oxysporum* (S-chl) f.sp*. cucumerinum* Owen, and *Fusarium graminearum* Schw at a concentration of 100 mg/L are listed in Table 1. Many of the synthesized compounds possessed moderate to high fungicidal activities. Thirteen compounds (**2**–**4**, **6**, **8**, **10**, **26**–**32**) showed inhibition rates exceeding 80% against *B. cinerea*, thirteen compounds (**2**–**4**, **6**, **8**, **10**, **26**–**32**) displayed more than 90% inhibition activities against *R. solani*, fifteen compounds (**2**–**4**, **6**–**10**, **26**–**32**) exhibited over 80% inhibition activities against *V. mali*, thirteen compounds (**2**–**4**, **6**, **8**, **10**, **26**–**32**) possessed over 80% inhibition activities against *T. cucumeris*, ten compounds (**3**, **6**, **10**, **26**–**32**) showed more than 80% effects against *F. oxysporum*, and eleven compounds (**2**, **3**, **6**, **10**, **26**–**32**) displayed more than 90% inhibition activity against *F. graminearum*. Among these compounds, compounds **3**, **6**, **26**–**32** showed broad spectra fungicidal activities against all tested phytopathogens with over 90% inhibition rates, especially, compounds **26**, **27**, **28**, **30**, **31** which showed 100% activities at a dosage of 100 mg/L. Compound **9** showed special activity against *V. mali*, displaying 84.30% inhibition activity, and showed low level of activities to *B. cinerea*, *F. oxysporum*, and *F. graminearum*. 

Compounds **1**–**4**, **6**–**11**, **26**–**32** were found to display good fungicidal activities and were chosen to do a rescreening. The bioassay data is summarized in Table 2. These compounds displayed growth-inhibitory activities with EC_50_ values ranges from 2.432–10.627, 2.182–11.024, 1.787–12.877, 1.638–10.253, 8.073–15.320, and 6.043–19.701 μg/mL against *B. cinerea*, *R. solani*, *V. mali*, *T. cucumeris*, *F. oxysporum*, and *F. graminearum*, respectively. Among them, compound **26** exhibited the highest activities, with EC_50_ values of 2.432, 2.182, 1.787, 1.638, 6.986, and 6.043 μg/mL against *B. cinerea*, *R. solani*, *V. mali*, *T. cucumeris*, *F. oxysporum*, and *F. graminearum*, respectively.

Based on the activities of pyrazole derivatives, structure-activity relationships can be discussed. The activities of the target compounds is attributed to the cyano group. Comparing the bioactivity between compounds with cyano groups and compounds containing COOEt groups, such as compounds **1** *versus* **3**, **2** *versus* **4**, **5** *versus* **6**, **7** *versus* **8**, **9** *versus* **10**, **15** *versus* **16**, **17** *versus* **18**, and **19** *versus* **20**, one can clearly see that all of these compounds but **9** and **10** obey the rule that the fungicide activity is improved when R^1^ was changed from a COOEt group to a cyano group. It was also found that the change of substituent on R^2^ could affect the fungicidal activity. Comparing the bioassay results of a number of pairs of compounds (**1** and **4**, **2** and **3**, **5** and **7**, **6** and **8**, **10** and **11**, **13** and **14**, *etc.*) which have the same R^1^ group, but different R^2^, one can confirm that when R^1^ is a COOEt group, the introduction of PhCH_2_ into R^2^ plays a positive effect on the activities of the compounds. By contrast, when R^1^ is a CN group, compounds with CH_3_ moieties showed higher activities than those with PhCH_2_ groups. 

In addition, the functional group diversity on R^3^ was also essential for the fungicidal activity of the title compounds. According to the data presented in Table 1 and Table 2, it can be observed that the introduction of a chlorine atom or fluorine atom on the substituted phenyl on R^3^ may improve the antifungal activity of the pyrazole derivatives. For instance, compound **12**, (R^3^ with a 4-carboxylphenyl moiety) showed lower activity than compounds **3** (R^3^ is 2,3,5,6-tetrafluorophenyl), **6** (R^3^ is 2,4,6-trichlorophenyl), and **26** (R^3^ is 4-trifluoromethyphenyl). Heterocyclic moieties were also introduced at R^3^, but the *N*-containing and *S*-containing heterocycles do not seem to enhance the activities of the title compounds, despite the change of position of the heteroatom or the introduction of halogen atoms. All of compounds **13**–**18**, **21**, **22** and **24** possessed low antifungal activities, for example.

**Table 1:** Fungicidal activities of compounds **1**–**32** at a concentration of 100 mg/L.

Comp.	Inhibitory Rates (%)					
	*B. cinerea*	*R. solani*	*V. mali*	*T. cucumeris*	*F. oxysporum*	*F. graminearum*
**1**	77.86 ^e^	79.30 ^d^	72.09 ^d^	67.87 ^d^	70.63 ^e^	51.25 ^g^
**2**	84.96 ^d^	98.02 ^b^	96.51 ^b^	88.78 ^c^	78.21 ^d^	91.00 ^c^
**3**	96.50 ^b^	98.44 ^b^	98.28 ^b^	98.87 ^b^	93.05 ^b^	98.05 ^b^
**4**	80.50 ^e^	93.38 ^c^	86.81 ^c^	85.73 ^c^	76.28 ^d^	73.00 ^d^
**5**	57.00 ^h^	66.98 ^f^	58.14 ^f^	50.12 ^g^	53.21 ^h^	67.07 ^e^
**6**	92.74 ^c^	97.25 ^b^	97.22 ^b^	96.13 ^b^	90.38 ^b^	98.05 ^b^
**7**	78.24 ^e^	79.19 ^d^	88.89 ^c^	60.62 ^ef^	71.79 ^e^	71.25 ^d^
**8**	85.00 ^d^	90.14 ^c^	94.19 ^b^	86.72 ^c^	71.79 ^e^	74.39 ^d^
**9**	47.36 ^j^	71.97 ^e^	84.30 ^c^	51.51 ^g^	35.90 ^k^	30.49 ^k^
**10**	92.14 ^c^	96.45 ^b^	95.83 ^b^	94.77 ^b^	84.56 ^c^	93.17 ^c^
**11**	62.00 ^g^	37.91 ^k^	72.22 ^d^	58.11 ^f^	36.54 ^k^	26.83 ^m^
**12**	49.50 ^j^	66.98 ^f^	48.26 ^g^	47.04 ^h^	32.05 ^l^	35.37 ^j^
**13**	52.44 ^i^	64.10 ^g^	65.23 ^e^	39.27 ^i^	65.79 ^g^	47.04 ^h^
**14**	40.22 ^k^	60.93 ^h^	21.62 ^j^	13.37 ^k^	48.03 ^i^	33.78 ^j^
**15**	65.49 ^g^	58.47 ^h^	66.22 ^e^	64.68 ^e^	68.42 ^f^	41.89 ^i^
**16**	41.40 ^k^	59.18 ^h^	34.12 ^i^	22.34 ^j^	36.18 ^k^	33.33 ^j^
**17**	33.86 ^m^	37.87 ^k^	37.87 ^i^	21.80 ^j^	35.53 ^k^	46.86 ^h^
**18**	34.42 ^m^	67.38 ^f^	52.05 ^g^	58.31 ^f^	42.11 ^j^	61.73 ^f^
**19**	36.19 ^l^	46.86 ^i^	41.89 ^h^	49.63 ^gh^	42.42 ^j^	46.97 ^h^
**20**	41.87 ^k^	62.21 ^h^	64.86 ^e^	57.73 ^f^	49.50 ^i^	52.33 ^g^
**21**	49.56 ^j^	65.23 ^fg^	51.35 ^g^	63.09 ^e^	42.17 ^j^	41.06 ^i^
**22**	36.74 ^l^	42.79 ^j^	33.78 ^i^	47.08 ^h^	36.01 ^k^	34.42 ^j^
**23**	41.32 ^k^	62.91 ^h^	43.24 ^h^	38.05 ^i^	44.74 ^j^	33.17 ^j^
**24**	65.49 ^g^	58.47 ^h^	66.22 ^e^	64.88 ^e^	68.42 ^f^	41.22 ^i^
**25**	42.42 ^k^	65.00 ^g^	39.19 ^h^	38.66 ^i^	67.07 ^f^	28.85 ^kl^
**26**	100.00 ^a^	100.00 ^a^	100.00 ^a^	100.00 ^a^	100.00 ^a^	100.00 ^a^
**27**	100.00 ^a^	100.00 ^a^	100.00 ^a^	100.00 ^a^	100.00 ^a^	100.00 ^a^
**28**	100.00 ^a^	100.00 ^a^	100.00 ^a^	100.00 ^a^	100.00 ^a^	100.00 ^a^
**29**	100.00 ^a^	100.00 ^a^	100.00 ^a^	100.00 ^a^	94.12^b^	100.00 ^a^
**30**	100.00 ^a^	100.00 ^a^	100.00 ^a^	100.00 ^a^	100.00 ^a^	100.00 ^a^
**31**	100.00 ^a^	100.00 ^a^	100.00 ^a^	100.00 ^a^	100.00 ^a^	100.00 ^a^
**32**	100.00 ^a^	100.00 ^a^	100.00 ^a^	100.00 ^a^	94.87 ^b^	92.48 ^c^

Notes: Values are means of three replicates; Letters a–m represent a significant difference at *p* = 0.05.

**Table 2:** Rescreening data of fungicidal activity of the synthesized compounds.

Comp.	EC_50_ (95% FL) (μg/mL)					
	*B. cinerea*	*R. solani*	*V. mali*	*T. cucumeris*	*F. oxysporum*	*F. graminearum*
**1**	10.627 (10.390–11.012)	11.024 (10.736–11.369)	12.822 (12.558–13.023)	--	15.320 (14.967–15.672)	--
**2**	8.073 (7.866–8.301)	6.987 (6.735–7.207)	4.622 (4.236–4.892)	9.515 (9.256–9.791)	13.588 (13.337–13.903)	15.435 (15.049–15.793)
**3**	5.848 (5.596–6.012)	6.043 (5.757–6.401)	3.738 (3.454–4.001)	5.707 (5.351–6.065)	9.515 (9.211–9.808)	14.793 (14.522–15.001)
**4**	9.891 (9.605–10.245)	8.991 (8.076–9.799)	5.707 (5.454–5.992)	10.253 (9.897–10.711)	13.859 (13.512–14.115)	19.162 (18.908–19.354)
**6**	7.233 (7.008–7.519)	6.519 (6.262–6.804)	3.757 (3.471–4.115)	6.096 (5.792–6.454)	9.699 (9.331–9.976)	15.002 (14.879–15.226)
**7**	10.641 (10.255–11.003)	11.877 (11.631–12.012)	6.043 (5.801–6.337)	--	15.053 (14.776–15.399)	19.701 (19.319–19.962)
**8**	8.221 (7.994–8.477)	8.037 (7.811–8.362)	4.987 (4.662–5.113)	9.891 (9.612–10.055)	14.793 (14.421–15.004)	19.114 (18.875–19.336)
**9**	--	13.327 (12.942–13.785)	5.314 (5.065–5.629)	--	--	--
**10**	7.493 (7.206–7.711)	6.748 (6.454–6.976)	4.455 (4.201–4.669)	6.519 (6.233–6.877)	10.668 (10.332–10.987)	15.320 (15.066–15.632)
**11**	--	--	12.877 (12.491–13.335)	--	---	--
**26**	2.432 (2.087–2.713)	2.182 (1.824–2.568)	1.787 (1.489–2.102)	1.638 (1.342–1.940)	6.986 (6.604–7.468)	6.043 (5.757–6.401)
**27**	3.742 (3.356–4.104)	3.501 (3.115–3.859)	1.919 (1.765–2.082)	2.383 (2.057–2.612)	8.073 (7.753–8.412)	10.266 (9.982–10.624)
**28**	3.870 (3.484–4.228)	3.619 (3.233–3.977)	2.432 (2.234–2.701)	2.570 (2.244–2.841)	8.221 (7.855–8.579)	10.688 (10.302–11.064)
**29**	4.843 (4.578–5.013)	5.897 (5.514–6.022)	3.330 (2.994–3.688)	3.181 (2.880–3.405)	9.171 (8.862–9.409)	12.080 (11.804–12.311)
**30**	4.006 (3.720–4.364)	4.148 (3.762–4.506)	2.760 (2.512–2.993)	2.989 (2.703–3.347)	8.359 (8.809–8.852)	11.088 (10.702–11.464)
**31**	4.297 (3.911–4.655)	4.455 (4.179–4.722)	2.989 (2.717–3.200)	3.083 (2.811–3.338)	8.665 (8.379–9.023)	11.329 (10.943–11.697)
**32**	4.994 (4.703–5.255)	6.097 (5.882–6.303)	3.458 (3.072–3.816)	3.390 (3.146–3.707)	9.339 (9.067–9.713)	15.053 (14.769–15.342)
*Carbendazole*	1.565 (1.328–1.779)	1.420 (1.266–1.637)	0.859 (0.692–1.006)	1.253 (1.007–1.469)	2.813 (2.582–3.011)	2.262 (2.007–2.522)

Moreover, two novel pyrazole derivatives **27**, **28** containing an amide moiety at the 5-position of the pyrazole ring were synthesized. The *in vitro* tests elucidated that the introduction of the amide group was beneficial for the improvement of the bioactivities. For example, when a 4-chlorophenyl carboxamide moiety was introduced to compound **3** to afford compound **27**, the antifungal activities were increased, as the EC_50_ values of **3** against *B. cinerea*, *R. solani*, *V. mali*, *T. cucumeris*, *F. oxysporum*, and *F. graminearum* were 5.848, 6.043, 3.738, 5.707, 9.515, and 14.793 μg/mL, respectively, while the EC_50_ values of **27** were 3.742, 3.501, 1.919, 2.383, 8.073, 10.266 μg/mL. Comparing compounds **4** and **28**, the same result that an amide moiety dramatically improved the potency of compound **28** was obtained. Furthermore, to search for the influence caused by the isothiocyanate structure, an isothiocyanatopropane moiety was introduced to compounds **3**, **4**, **6**, and **8**, thus generating compounds **30**, **29**, **31**, and **32**, respectively. Among these compounds, **30**, **29**, **31** and **32** which contain an isothiocyanate group were more active than the compounds without such a group, especially compound **29**, which EC_50_ value of against *T. cucumeris* was 3.181 μg/mL, much lower than compound **4** with an EC_50_ value of 10.253 μg/mL. Due to the lack of a wide range of compounds with amide and isothiocyanate moieties, more detailed structure-activity discussions of the fungicidal activity is almost impossible, but the findings mentioned above suggested that amide and isothiocyanate moieties are essential for obtaining interesting fungicidal activity for the designed compounds and pyrazole derivatives containing amide and isothiocyanate moieties are worthy of further study.

## 3. Experimental Section

### 3.1. General Information

^1^H-NMR and ^13^C-NMR spectra were recorded on a Bruker AV500 spectrometer (Bruker, Bremerhaven, Germany) in CDCl_3_, CD_3_COCD_3_, pyridine or DMSO-*d*_6_ solution with TMS (tetramethylsilane) as the internal standard. Chemical shift values (δ) are given in parts per million (ppm). HR-ESI-MS spectra were carried out using a Bruker apex-ultra 7.0 T spectrometer. The measurement of melting points was conducted on an X-4 binocular microscope melting point apparatus (Beijing Tech Instruments Co., Beijing, China). All chemical reagents and solvent were of analytical grade. Silica gel for Thin Layer Chromatography (TLC) and Column Chromatography (CC) was purchased from Qingdao Haiyang Chemical Co. Ltd. (Qingdao, China).

### 3.2. Chemical Synthesis

All anhydrous solvents were dried and purified by standard techniques before use. The synthetic route is given in Scheme 1.

#### 3.2.1. General Procedure for the Synthesis of Compounds **3a**–**d**

A previously described method was used to synthesize compound **3a** [33]. In brief, powdered KOH (0.3 mol, 16.83 g) was added to a solution of CH_3_CN (125 mL) and cooled to 0 °C by means of an ice bath. Then malononitrile (0.15 mol, 9.91 g) was added dropwise, and after the color of the reaction mixture became light yellow, CS_2_ (0.15 mol, 11.42 g) was added in portions. The system was kept at room temperature for 2 h. The final slurry was filtered under vacuum, filtered and washed with ether (3 × 100 mL), dried, and then transferred to a mixture of methanol–water (5:1 by volume) placed in a 250 mL round-bottomed flask equipped with a mechanical stirrer. While stirring at room temperature, benzyl chloride (0.3 mol, 37.97 g) was added dropwise from a dropping funnel into the mixture. After the addition was completed, the system was continuously stirred for 3 h. The mixture was filtered and washed with ether (3 × 100 mL) to obtain a white solid. A pure sample can be achieved by recrystallization from ethanol. Compounds **3b**–**d** were synthesized by following the procedure of intermediate **2a**–**b** using NCCH_2_COOEt replacing malononitrile and dimethyl sulfate in place of benzyl chloride. 

#### 3.2.2. General Procedure for the Preparation of Compounds **1**–**26**

In a 250 mL three-necked round-bottom flask equipped with a magnetic stirrer and a reflux condenser, anhydrous ethanol (30 mL), different hydrazines (0.05 mol), and intermediate compounds **3a**–**d** were added. Then the reaction mixture was heated to reflux and stirred till the reaction was complete, as monitored by TLC. After completion, the mixture was filtered to obtain crude target compounds. The purification procedure of compounds **9**–**25** was recrystallization from isopropanol, the residue was purified through column chromatography using petroleum ether and ether as eluent to get products **1**–**8**, **26** [34].

*Ethyl 5-amino-3-(methylthio)-1-(2,3,5,6-tetrafluorophenyl)-1H-pyrazole-4-*carboxylate (**1**). Yield, 92%; brown solid; m.p. 140–143 °C; ^1^H-NMR (500 MHz, CD_3_COCD_3_) δ: 7.80–7.87 (1H, m), 6.47 (2H, s), 4.29 (2H, q, *J* = 7.1 Hz), 2.42 (3H, s), 1.35 (3H, t, *J* = 7.1 Hz); ^13^C-NMR (125 MHz, CD_3_COCD_3_) δ: 163.3, 153.9, 151.8, 147.3, 145.4, 144.8, 142.9, 108.1, 107.9, 107.7, 92.4, 59.2, 14.0, 12.0; HR-MS (ESI): *m/z* calcd for C_13_H_11_N_3_O_2_F_4_S ([M+H]^+^), 350.0581; found, 350.0577.

*5-Amino-3-(benzylthio)-1-(2,3,5,6-tetrafluorophenyl)-1H-pyrazole-4-carbonitrile* (**2**). Yield, 89%; brown solid; m.p. 155–158 °C; ^1^H-NMR (500 MHz, DMSO) δ: 8.20–8.27 (1H, m), 7.36 (2H, d, *J* = 7.5 Hz), 7.35 (2H, s),7.31 (2H, t, *J* = 7.2 Hz), 7.26 (1H, t, *J* = 7.1 Hz), 4.26 (2H, s); ^13^C-NMR (125 MHz, DMSO) δ: 155.8, 150.8, 147.6, 145.7, 145.3, 143.3, 138.1, 129.9, 129.3, 128.3, 114.3, 110.0, 109.8, 109.6, 73.7, 35.7; HR-MS (ESI): *m/z* calcd for C_17_H_10_N_4_F_4_S ([M+H]^+^), 379.0635; found, 379.0631.

*5-Amino-3-(methylthio)-1-(2,3,5,6-tetrafluorophenyl)-1H-pyrazole-4-carbonitrile* (**3**). Yield, 90%; brown solid; m.p. 134–138 °C; ^1^H-NMR (500 MHz, DMSO) δ: 8.21–8.29 (1H, m), 7.37 (2H, s), 2.49 (3H, s); ^13^C-NMR (125 MHz, DMSO) δ: 155.9, 152.4, 147.7, 145.7, 145.6, 145.4, 145.2, 143.4, 143.3, 114.4, 110.0, 109.8, 109.6, 72.7, 13.9; HR-MS (ESI): *m/z* calcd for C_11_H_6_N_4_F_4_S ([M+H]^+^), 303.0322; found, 303.0310.

*Ethyl 5-amino-3-(benzylthio)-1-(2,3,5,6-tetrafluorophenyl)-1H-pyrazole-4-carboxylate* (**4**). Yield, 92%; brown solid; m.p. 138–142 °C; ^1^H-NMR (500 MHz, CD_3_COCD_3_) δ: 7.81–7.85 (1H, m), 7.47 (2H, d, *J* = 7.3 Hz), 7.33 (2H, t, *J* = 7.4 Hz), 7.27 (1H, t, *J* = 7.4 Hz), 6.51 (2H, s), 4.29 (2H, s), 4.27 (2H, q, *J* = 7.1 Hz), 1.31 (3H, t, *J* = 7.1 Hz); ^13^C-NMR (125 MHz, CD_3_COCD_3_) δ: 163.2, 153.8, 150.9, 147.4, 145.4, 144.9, 142.9, 138.1, 129.2, 128.3, 127.0, 108.1, 108.0, 107.8, 92.3, 59.3, 33.7, 14.0; HR-MS (ESI): *m/z* calcd for C_19_H_15_N_3_O_2_F_4_S ([M+H]^+^), 426.0894; found, 426.0903.

*Ethyl 5-amino-3-(methylthio)-1-(2,4,6-trichlorophenyl)-1H-pyrazole-4-carboxylate* (**5**). Yield, 95%; white solid; m.p. 170–173 °C; ^1^H-NMR (500 MHz, DMSO) δ: 7.96 (2H, s), 6.59 (2H, s), 4.24 (2H, q, *J* = 7.1 Hz), 2.35 (3H, s), 1.31 (3H, t, *J* = 7.1 Hz); ^13^C-NMR (125 MHz, DMSO) δ: 163.4, 153.1, 150.5, 136.4, 136.3, 132.3, 129.5, 91.5, 59.4, 15.0, 12.8; HR-MS (ESI): *m/z* calcd for C_13_H_12_N_3_O_2_SCl_3_ ([M+H]^+^), 379.9789; found, 379.9791.

*5-Amino-3-(methylthio)-1-(2,4,6-trichlorophenyl)-1H-pyrazole-4-carbonitrile* (**6**). Yield, 95%; white solid; m.p. 184–187 °C; ^1^H-NMR (500 MHz, CDCl_3_) δ: 7.52 (2H, s), 4.61 (2H, s), 2.54 (3H, s); ^13^C-NMR (125 MHz, CDCl_3_) δ: 152.9, 152.1, 138.0, 136.8, 130.8, 129.5, 113.7, 75.6, 14.5; HR-MS (ESI): *m/z* calcd for C_11_H_7_N_4_SCl_3_ ([M+H]^+^), 332.9530; found, 332.9519.

*Ethyl 5-amino-3-(benzylthio)-1-(2,4,6-trichlorophenyl)-1H-pyrazole-4-carboxylate* (**7**). Yield, 94%; white solid; m.p. 163–165 °C; ^1^H NMR (500 MHz, CDCl_3_) δ: 7.50 (2H, s), 7.42 (2H, d, *J* = 7.2 Hz), 7.28 (2H, t, *J* = 7.1 Hz), 7.22 (1H, t, *J* = 7.1 Hz), 5.23 (2H, s), 4.28 (2H, q, *J* = 7.1 Hz), 4.26 (2H, s), 1.35 (3H, t, *J* = 7.1 Hz); ^13^C-NMR (125 MHz, CDCl_3_) δ: 164.2, 152.0, 150.1, 137.7, 137.1, 136.6, 131.2, 129.3, 129.2, 128.4, 127.1, 93.5, 60.0, 34.6, 14.5; HR-MS (ESI): *m/z* calcd for C_19_H_16_N_3_O_2_SCl_3_ ([M+H]^+^), 456.0102; found, 456.0122.

*5-Amino-3-(benzylthio)-1-(2,4,6-trichlorophenyl)-1H-pyrazole-4-carbonitrile* (**8**). Yield, 96%; white solid; m.p. 162–164 °C; ^1^H-NMR (500 MHz, CDCl_3_) δ: 7.51 (2H, s), 7.36 (2H, d, *J* = 7.2 Hz), 7.26 (2H, t, *J* = 7.1 Hz), 7.23 (1H, t, *J* = 7.1 Hz), 4.42 (2H, s), 4.26 (2H, s); ^13^C-NMR (125 MHz, CDCl_3_) δ: 152.4, 150.3, 138.1, 137.3, 136.7, 130.7, 129.6, 129.5, 128.8, 127.7, 113.4, 36.9; HR-MS (ESI): *m/z* calcd for C_17_H_11_N_4_SCl_3_ ([M+H]^+^), 408.9843; found, 408.9844.

*4-(5-Amino-3-(benzylthio)-4-cyano-1H-pyrazol-1-yl)benzoic acid* (**9**). Yield, 90%; brown solid; m.p. 231–232 °C; ^1^H-NMR (500 MHz, DMSO) δ: 13.23 (1H, s), 8.15 (2H, d, *J* = 8.7 Hz), 7.72 (2H, d, *J* = 8.6 Hz), 7.47 (2H, d, *J* = 7.2 Hz), 7.40 (2H, t, *J* = 7.5 Hz), 7.34 (1H, t, *J* = 7.3 Hz), 7.11 (2H, s), 4.39 (2H, s); ^13^C-NMR (125 MHz, DMSO) δ: 167.4, 153.4, 149.3, 141.7, 138.3, 131.6, 130.5, 129.9, 129.3, 128.2, 124.2, 114.6, 75.8, 35.8; HR-MS (ESI): *m/z* calcd for C_18_H_14_N_4_O_2_S ([M+H]^+^), 351.0910; found, 351.0900.

*4-(5-Amino-3-(benzylthio)-4-(ethoxycarbonyl)-1H-pyrazol-1-yl)benzoic acid* (**10**). Yield, 91%; brown solid; m.p. 226–228 °C; ^1^H-NMR (500 MHz, DMSO) δ: 13.15 (1H, s), 8.13 (2H, d, *J* = 8.7 Hz), 7.75 (2H, d, *J* = 8.7 Hz), 7.48 (2H, d, *J* = 7.2 Hz), 7.36 (2H, t, *J* = 7.5 Hz), 7.29 (1H, t, *J* = 7.3 Hz), 6.66 (2H, s), 4.31 (2H, s), 4.24 (2H, q, *J* = 7.1 Hz), 1.29 (3H, t, *J* = 7.1 Hz); ^13^C-NMR (125 MHz, DMSO) δ: 167.5, 164.0, 152.2, 149.7, 142.2, 138.9, 131.6, 130.1, 129.9, 129.2, 127.9, 123.4, 93.8, 60.2, 34.2, 15.3; HR-MS (ESI): *m/z* calcd for C_20_H_19_N_3_O_4_S ([M+H]^+^), 398.1169; found, 398.1173.

*4-(5-Amino-4-(ethoxycarbonyl)-3-(methylthio)-1H-pyrazol-1-yl)benzoic acid* (**11**). Yield, 90%; brown solid; m.p. > 260 °C; ^1^H-NMR (500 MHz, DMSO) δ: 13.11 (1H, s), 8.11 (2H, d, *J* = 8.7 Hz), 7.75 (2H, d, *J* = 8.7 Hz), 6.62 (2H, s), 4.26 (2H, q, *J* = 7.1 Hz), 2.46 (3H, s), 1.32 (3H, t, *J* = 7.1 Hz); ^13^C-NMR (125 MHz, DMSO) δ: 167.5, 164.1, 152.4, 150.7, 142.3, 131.5, 129.9, 123.6, 94.0, 60.2, 15.3, 13.3; HR-MS (ESI): *m/z* calcd for C_14_H_15_N_3_O_4_S ([M+H]^+^), 322.0856; found, 322.0845.

*4-(5-Amino-4-cyano-3-(methylthio)-1H-pyrazol-1-yl)benzoic acid* (**12**). Yield, 92%; brown solid; m.p. > 260 °C; ^1^H-NMR (500 MHz, DMSO+Benzene) δ: 13.06 (1H, s), 8.14 (2H, d, *J* = 8.2 Hz), 7.67 (2H, d, *J* = 8.1 Hz), 7.00 (2H, s), 2.49 (3H, s); ^13^C-NMR (125 MHz, DMSO) δ: 167.5, 153.5, 150.8, 141.8, 131.5, 130.4, 124.3, 114.7, 74.7, 14.1; HR-MS (ESI): *m/z* calcd for C_12_H_10_N_4_O_2_S ([M+H]^+^), 275.0587; found, 275.0597.

*Ethyl 5-amino-3-(benzylthio)-1-(thiophene-2-carbonyl)-1H-pyrazole-4-carboxylate* (**13**). Yield, 95%; white solid; m.p. > 260 °C; ^1^H-NMR (500 MHz, CDCl_3_) δ: 8.30 (1H, d, *J* = 3.9 Hz), 7.76 (1H, d, *J* = 3.8 Hz), 7.48 (2H, d, *J* = 7.3 Hz), 7.34 (2H, t, *J* = 7.4 Hz), 7.29 (1H, t, *J* = 7.3 Hz), 7.17 (1H, t, *J* = 4.5 Hz), 4.48 (2H, s), 4.30 (2H, q, *J* = 7.1 Hz), 1.35 (3H, t, *J* = 7.1 Hz); ^13^C-NMR (125 MHz, CDCl_3_) δ: 164.2, 162.0, 155.9, 154.3, 138.4, 138.1, 137.1, 132.5, 129.6, 129.0, 127.8, 127.4, 93.3, 60.6, 35.6, 14.8; HR-MS (ESI): *m/z* calcd for C_18_H_17_N_3_O_3_S_2_ ([M+H]^+^), 388.0784; found, 388.0773.

*Ethyl 5-amino-3-(methylthio)-1-(thiophene-2-carbonyl)-1H-pyrazole-4-carboxylate* (**14**). Yield, 96%; white solid; m.p. > 260 °C; ^1^H-NMR (500 MHz, DMSO) δ: 8.32 (1H, d, *J* = 3.9 Hz), 8.21 (1H, d, *J* = 5.0 Hz), 7.33 (1H, t, *J* = 4.5 Hz), 4.27 (2H, q, *J* = 7.1 Hz), 2.59 (3H, s), 1.32 (3H, t, *J* = 7.1 Hz); ^13^C-NMR (125 MHz, DMSO) δ: 163.5, 161.5, 155.8, 155.0, 140.1, 139.1, 132.6, 128.3, 92.7, 60.5, 15.3, 14.1; HR-MS (ESI): *m/z* calcd for C_12_H_13_N_3_O_3_S_2_ ([M+H]^+^), 312.0471; found, 312.0464.

*5-Amino-1-(5-chlorothiophene-2-carbonyl)-3-(methylthio)-1H-pyrazole-4-carbonitrile* (**15**). Yield, 93%; white solid; m.p. > 260 °C; ^1^H-NMR (500 MHz, Py) δ: 9.05 (2H, s), 8.18 (1H, d, *J* = 4.2 Hz), 7.10 (1H, d, *J* = 4.1 Hz), 2.62 (3H, s); ^13^C-NMR (125 MHz, Py) δ: 160.1, 157.2, 155.1, 142.9, 138.0, 130.3, 127.1, 113.0, 74.6, 13.7; HR-MS (ESI): *m/z* calcd for C_10_H_7_N_4_OS_2_Cl ([M+H]^+^), 298.9793; found, 298.9799.

*Ethyl 5-amino-1-(5-chlorothiophene-2-carbonyl)-3-(methylthio)-1H-pyrazole-4-carboxylate* (**16**). Yield, 92%; white solid; m.p. > 260 °C; ^1^H-NMR (500 MHz, DMSO) δ: 8.14 (1H, d, *J* = 4.3 Hz), 7.39 (1H, d, *J* = 4.3 Hz), 4.26 (2H, q, *J* = 7.1 Hz), 2.58 (3H, s), 1.32 (3H, t, *J* = 7.1 Hz); ^13^C-NMR (125 MHz, DMSO) δ: 163.4, 160.1, 155.6, 155.4, 142.4, 138.7, 130.0, 128.2, 92.9, 60.6, 15.3, 14.2; HR-MS (ESI): *m/z* calcd for C_12_H_12_N_3_O_3_S_2_Cl ([M+H]^+^), 346.0071; found, 346.0081.

*Ethyl 5-amino-1-(5-bromothiophene-2-carbonyl)-3-(methylthio)-1H-pyrazole-4-carboxylate* (**17**). Yield, 94%; white solid; m.p. > 260 °C; ^1^H-NMR (500 MHz, DMSO) δ: 8.06 (1H, d, *J* = 4.2 Hz), 7.47 (1H, d, *J* = 4.2 Hz), 4.26 (2H, q, *J* = 7.1 Hz), 2.58 (3H, s), 1.32 (3H, t, *J* = 7.1 Hz); ^13^C-NMR (125 MHz, DMSO) δ: 163.4, 160.0, 155.5, 155.4, 139.2, 132.9, 131.5, 127.5, 92.9, 60.6, 15.3, 14.2; HR-MS (ESI): *m/z* calcd for C_12_H_12_N_3_O_3_S_2_Br ([M+H]^+^), 389.9576; found, 389.9575. 

*5-Amino-1-(5-bromothiophene-2-carbonyl)-3-(methylthio)-1H-pyrazole-4-carbonitrile* (**18**). Yield, 95%; white solid; m.p. > 260 °C; ^1^H-NMR (500 MHz, DMSO) δ: 8.18 (2H, s), 8.09 (1H, d, *J* = 4.2 Hz), 7.46 (1H, d, *J* = 4.3 Hz), 2.70 (3H, s); ^13^C-NMR (125 MHz, DMSO) δ: 159.6, 156.8, 155.0, 139.2, 132.8, 131.4, 126.9, 113.0, 73.4, 13.8; HR-MS (ESI): *m/z* calcd for C_10_H_7_N_4_OS_2_Br ([M+H]^+^), 342.9172; found, 342.9175.

*Ethyl 1-acetyl-5-amino-3-(methylthio)-1H-pyrazole-4-carboxylate* (**19**). Yield, 90%; white solid; m.p. 110–113 °C; ^1^H-NMR (500 MHz, CD_3_COCD_3_) δ: 4.28 (2H, q, *J* = 7.1 Hz), 2.59 (3H, s), 2.49 (3H, s), 1.35 (3H, t, *J* = 7.1 Hz); ^13^C-NMR (125 MHz, CD_3_COCD_3_) δ: 174.5, 164.8, 156.1, 154.8, 94.0, 61.1, 23.9, 15.5, 13.5; HR-MS (ESI): *m/z* calcd for C_9_H_13_N_3_O_3_S ([M+H]^+^), 244.0710; found, 244.0711.

*1-Acetyl-5-amino-3-(methylthio)-1H-pyrazole-4-carbonitrile* (**20**). Yield, 93%; white solid; m.p. 212–215 °C; ^1^H-NMR (500 MHz, DMSO) δ: 8.06 (2H, s), 2.56 (3H, s), 2.55 (3H, s); ^13^C-NMR (125 MHz, DMSO) δ: 173.2, 156.1, 153.2, 113.8, 73.1, 24.1, 13.4; HR-MS (ESI): *m/z* calcd for C_7_H_8_N_4_OS ([M+H]^+^), 197.0449; found, 197.0452.

*5-Amino-3-(methylthio)-1-nicotinoyl-1H-pyrazole-4-carbonitrile* (**21**). Yield, 86%; white solid; m.p. 182–185 °C; ^1^H-NMR (500 MHz, DMSO) δ: 9.14 (1H, s), 8.81 (1H, d, *J* = 3.6 Hz), 8.39 (1H, d, *J* = 8.1 Hz), 8.31 (2H, s), 7.60 (1H, dd, *J* = 4.9 Hz, *J* = 7.9 Hz), 2.48 (3H, s); ^13^C-NMR (125 MHz, DMSO) δ: 167.9, 157.4, 154.3, 153.7, 151.8, 139.3, 129.5, 123.9, 113.7, 73.2, 13.4; HR-MS (ESI): *m/z* calcd for C_1__1_H_9_N_5_OS ([M+H]^+^), 260.0558; found, 260.0561.

*5-Amino-3-(benzylthio)-1-nicotinoyl-1H-pyrazole-4-carbonitrile* (**22**). Yield, 88%; white solid; m.p. 201–205 °C; ^1^H-NMR (500 MHz, Py) δ: 9.50 (1H, s), 8.89 (1H, d, *J* = 3.2 Hz), 8.39 (1H, d, *J* = 8.0 Hz), 7.43 (2H, d, *J* = 7.5 Hz), 7.41 (1H, dd, *J* = 4.9 Hz, *J* = 7.9 Hz), 7.32 (2H, t, *J* = 7.5 Hz), 7.25 (1H, t, *J* = 7.3 Hz), 4.38 (2H, s); ^13^C-NMR (125 MHz, Py) δ: 167.8, 157.4, 153.3, 153.2, 151.9, 138.5, 137.6, 129.4, 129.2, 128.8, 127.7, 123.0, 113.3, 74.4, 34.9; HR-MS (ESI): *m/z* calcd for C_1__7_H_13_N_5_OS ([M+H]^+^), 336.0871; found, 336.0874.

*5-Amino-3-(benzylthio)-1-(4-hydroxybenzoyl)-1H-pyrazole-4-carbonitrile* (**23**). Yield, 89%; white solid; m.p. 202–204 °C; ^1^H-NMR (500 MHz, Py) δ: 12.83 (1H, s), 9.18 (2H, s), 8.33 (2H, d, *J* = 8.8 Hz), 7.49 (2H, d, *J* = 7.3 Hz), 7.28 (2H, d, *J* = 7.5 Hz), 7.22 (2H, d, *J* = 8.8 Hz), 7.21 (1H, t, *J* = 5.2 Hz), 4.46 (2H, s); ^13^C-NMR (125 MHz, Py) δ: 166.9, 162.2, 156.2, 150.2, 136.0, 133.2, 127.7, 127.1, 126.0, 121.4, 114.0, 112.1, 72.7, 33.4; HR-MS (ESI): *m/z* calcd for C_18_H_14_N_4_O_2_S ([M+H]^+^), 351.0900; found, 351.0892.

*5-Amino-1-isonicotinoyl-3-(methylthio)-1H-pyrazole-4-carbonitrile* (**24**). Yield, 92%; white solid; m.p. > 260 °C; ^1^H-NMR (500 MHz, Py) δ: 9.10 (2H, s), 8.91 (2H, d, *J* = 5.8 Hz), 7.99 (2H, d, *J* = 5.8 Hz), 2.45 (3H, s); ^13^C-NMR (125 MHz, Py) δ: 167.9, 157.8, 155.1, 150.4, 140.2, 124.2, 113.1, 13.2; HR-MS (ESI): *m/z* calcd for C_11_H_9_N_5_OS ([M+H]^+^), 260.0558; found, 260.0561.

*5-Amino-1-(3-methoxybenzoyl)-3-(methylthio)-1H-pyrazole-4-carbonitrile* (**25**). Yield, 87%; white solid; m.p. 173–174 °C; ^1^H-NMR (500 MHz, DMSO) δ: 8.25 (2H, s), 7.61 (1H, s), 7.60 (1H, d, *J* = 7.5 Hz), 7.48 (1H, t, *J* = 7.9 Hz), 7.26 (1H, d, *J* = 7.0 Hz), 3.84 (3H, s), 2.49 (3H, s); ^13^C-NMR (125 MHz, DMSO) δ: 169.0, 159.3, 157.6, 153.8, 134.2, 130.0, 124.0, 119.9, 116.6, 113.8, 73.1, 56.3, 13.5; HR-MS (ESI): *m/z* calcd for C_13_H_12_N_4_O_2_S ([M+H]^+^), 289.0754; found, 289.0763.

*5-Amino-3-(methylthio)-1-(4-(trifluoromethyl)phenyl)-1H-pyrazole-4-carbonitrile* (**26**). Yield, 90%; brown solid; m.p. 95–97 °C; ^1^H-NMR (500 MHz, CDCl_3_) δ: 7.79 (2H, d, *J* = 8.4 Hz), 7.68 (2H, d, *J* = 8.3 Hz), 4.67 (2H, s), 2.58 (3H, s); ^13^C-NMR (125 MHz, CDCl_3_) δ: 151.6, 151.2, 140.3, 130.8, 130.6, 127.6, 127.5, 124.0, 113.4, 14.4; HR-MS (ESI): *m/z* calcd for C_12_H_9_N_4_F_3_S ([M+H]^+^), 299.0573; found, 299.0567.

#### 3.2.3. General Procedure for Compounds **27**–**28**

For the preparation of compounds **27**–**28**, 4-fluorobenzoic acid (1 mmol, 140.1 mg) or 3-chlorobenzoic acid (1 mmol, 156.6 mg) was added to a well-stirred solution of DCC (1.2 mmol, 247.6 mg) in DCM. After the mixture became dark, compound **3** or **4** and DMAP (0.24 mmol, 29.3 mg) were added, stirred at room temperature monitored by TLC, until the reaction finished [35]. The contents of flask were filtered, the filtrate was collected, the solvent was removed under reduced pressure and the residue was purified on silica gel, eluting with a mixture of petroleum ether and ethyl acetate (2:1, by volume). 

*N-(4-Cyano-3-(methylthio)-1-(2,3,5,6-tetrafluorophenyl)-1H-pyrazol-5-yl)-4-fluorobenzamide* (**27**). Yield, 80%; brown solid; m.p. 121–123 °C; ^1^H-NMR (500 MHz, CDCl_3_) δ: 9.58 (1H, s), 7.84 (2H, q, *J* = 7.1 Hz), 7.14–7.20 (1H, m), 7.09 (2H, t, *J* = 8.6 Hz), 2.61 (3H, s); ^13^C-NMR (125 MHz, CDCl_3_) δ: 165.1, 164.7, 154.2, 153.9, 147.3, 145.3, 144.5, 142.6, 131.0, 129.8, 116.4, 114.2, 108.2, 108.0, 107.8, 14.3; HR-MS (ESI): *m/z* calcd for C_18_H_9_N_4_OF_5_S ([M+H]^+^), 425.0490; found, 425.0499.

*Ethyl 3-(benzylthio)-5-(3-chlorobenzamido)-1-(2,3,5,6-tetrafluorophenyl)-1H-pyrazole-4-carboxylate* (**28**). Yield, 85%; white solid; m.p. 144–147 °C; ^1^H-NMR (500 MHz, CDCl_3_) δ: 10.23 (1H, s), 7.83 (1H, t, *J* = 1.8 Hz), 7.73 (1H, d, *J* = 7.7 Hz), 7.55 (1H, d, *J* = 8.0 Hz), 7.43 (1H, s), 7.42 (2H, d, *J* = 7.8 Hz), 7.32 (2H, t, *J* = 7.3 Hz), 7.27 (1H, t, *J* = 7.3 Hz), 7.13–7.20 (1H, m), 4.37 (2H, q, *J* = 7.1 Hz), 4.35 (2H, s), 1.38 (3H, t, *J* = 7.1 Hz); ^13^C-NMR (125 MHz, CDCl_3_) δ: 163.2, 161.9, 150.0, 144.3, 136.0, 134.4, 132.7, 132.2, 129.3, 128.3, 127.5, 127.2, 126.4, 124.5, 105.7, 105.5, 105.3, 99.5, 60.3, 33.7, 13.3; HR-MS (ESI): *m/z* calcd for C_26_H_18_N_3_O_3_F_4_SCl ([M+H]^+^), 564.0766; found, 564.0766.

#### 3.2.4. General Procedure for Compounds **29**–**32**

A total of 1 mmol of 1,3,4-substituted-5-amino pyrazoles (**3**, **4**, **6** or **8**) and potassium hydroxide powder (1 mmol, 561.1 mg) were mixed with THF (20 mL) of in a 100 mL round-bottom flask, then 1,3-dibromopropane (1 mmol, 201.9 mg) was added dropwise to the mixture. After 6 h of stirring at room temperature, sodium azide (1.5 mmol, 975.2 mg) was added and heated at 45 °C for 3 h. Then, triphenylphosphine (1 mmol, 262.3 mg) and carbon disulfide (1.5 mmol, 114.2 mg) were added, stirred at room temperature until the reaction was completed [34]. The final slurry was filtered, the filtrate was evaporated under reduced pressure, and the residue was purified through column chromatography using petroleum ether and ether as eluent to afford **29**–**32**.

*Ethyl 3-(benzylthio)-5-(3-isothiocyanatopropylamino)-1-(2,3,5,6-tetrafluorophenyl)-1H-pyrazole-4-carboxylate* (**29**). Yield, 78%; white solid; m.p. 92–95 °C; ^1^H-NMR (500 MHz, CDCl_3_) δ: 7.40 (2H, d, *J* = 7.4 Hz), 7.30 (2H, t, *J* = 7.3 Hz), 7.24 (1H, t, *J* = 7.2 Hz), 6.59–6.64 (1H, m), 4.29 (2H, q, *J* = 7.1 Hz), 4.26 (2H, s), 3.37 (2H, t, *J* = 6.1 Hz), 3.04 (2H, t, *J* = 6.1 Hz), 1.93–1.98 (2H, m), 1.35 (3H, t, *J* = 7.1 Hz); ^13^C-NMR (125 MHz, CDCl_3_) δ: 165.0, 155.6, 151.9, 144.9, 143.8, 142.6, 137.7, 129.7, 128.9, 127.6, 113.0, 108.1, 107.9, 107.7, 95.2, 60.6, 42.6, 34.8, 32.8, 30.0, 14.8; HR-MS (ESI): *m/z* calcd for C_23_H_20_N_4_O_2_F_4_S_2_ ([M+H]^+^), 525.0994; found, 525.0997.

*5-(3-Isothiocyanatopropylamino)-3-(methylthio)-1-(2,3,5,6-tetrafluorophenyl)-1H-pyrazole-4-carbonitrile* (**30**). Yield, 76%; white solid; m.p. 105–108 °C; ^1^H-NMR (500 MHz, CDCl_3_) δ: 7.27–7.34 (1H, m), 4.06 (2H, t, *J* = 7.6 Hz), 3.16 (2H, t, *J* = 6.8 Hz), 2.56 (3H, s), 2.37–2.44 (2H, m); ^13^C-NMR (125 MHz, CDCl_3_) δ: 153.7, 152.9, 147.5, 145.5, 144.9, 142.6, 113.2, 111.5, 108.5, 108.3, 108.1, 75.8, 53.9, 31.6, 29.9, 14.0; HR-MS (ESI): *m/z* calcd for C_15_H_11_N_5_F_4_S_2_ ([M+H]^+^), 402.0422; found, 402.0426.

*5-(3-Isothiocyanatopropylamino)-3-(methylthio)-1-(2,4,6-trichlorophenyl)-1H-pyrazole-4-carbonitrile* (**31**). Yield, 76%; white solid; m.p. 90–94 °C; ^1^H-NMR (500 MHz, CDCl_3_) δ: 7.21 (2H, s), 3.71 (4H, t, *J* = 7.6 Hz), 2.25 (3H, s), 2.05–2.11 (2H, m); ^13^C-NMR (125 MHz, CDCl_3_) δ: 154.9, 152.6, 137.3, 136.9, 129.5, 128.8, 117.4, 114.4, 74.3, 60.6, 53.5, 18.1, 14.3; HR-MS (ESI): *m/z* calcd for C_15_H_12_N_5_S_2_Cl_3_ ([M+H]^+^), 431.9583; found, 431.9577.

*3-(Benzylthio)-5-(3-isothiocyanatopropylamino)-1-(2,4,6-trichlorophenyl)-1H-pyrazole-4-carbonitrile* (**32**). Yield, 74%; white solid; m.p. 87–90 °C; ^1^H-NMR (500 MHz, CDCl_3_) δ: 7.45 (2H, s), 7.35 (2H, d, *J* = 7.2 Hz), 7.27 (2H, t, *J* = 7.5 Hz), 7.22 (1H, t, *J* = 7.3 Hz), 5.62 (1H, s), 4.23 (2H, s), 3.95 (4H, t, *J* = 7.4 Hz), 2.29–2.35 (2H, m); ^13^C-NMR (125 MHz, CDCl_3_) δ: 155.0, 151.0, 137.4, 137.0, 131.2, 129.5, 128.9, 128.8, 128.3, 127.6, 114.4, 76.1, 58.2, 53.7, 36.8, 18.2; HR-MS (ESI): *m/z* calcd for C_21_H_16_N_5_S_2_Cl_3_ ([M+H]^+^), 507.9880; found, 507.9883. 

### 3.3. Bioassay of Antifungal Activity

#### 3.3.1. Preparation of Tested Fungal Pathogens

The fungal pathogens *B. cinerea*, *R. solani*, *V. mali*, *T. cucumeris*, *F. oxysporum*, and *F. graminearum* were provided by Northwest A&F University (Yangling, China). *R. solani*, *V. mali*, *T. cucumeris*, *F. oxysporum*, and *F. graminearum* were activated for 1 week at 25 °C on potato dextrose agar (PDA) media, while *B. cinerea* was cultured at 20 °C on the same medium after being retrieved from the storage tube. A punch was used to make agar discs with mycelium (4 mm in diameter).

#### 3.3.2. Fungicidal Activity Assay

For initial screening of fungicidal activities of title compounds, test compounds were dissolved in 1000 mg/L and mixed with sterile molten potato dextrose agar (PDA) to obtain final concentration of 100 mg/L. In the terms of rescreening antifungal activity, two-fold serial-dilution technique was used to prepare stock solution of compounds **1**–**32** [36]. For this purpose, tested samples dissolved in acetone or DMSO at a concentration of 2000 mg/L to form the initial dilution. Then, the solution was diluted with 0.1% Tween-20 in water to obtain a set of eight dilutions of test compounds. After completion, 9 mL of the PDA inoculated broth was taken in a graduated test tube with stopper and the prepared solution (1 mL) was added to it to form a mixture containing tested compound concentration of 100, 50, 25, 12.5, 6.25, 3.13, 1.56, and 0.78 mg/L, with the final concentration of acetone or DMSO lower than 1% (*v*/*v*). Then, mixture was poured into culture dish (9 cm in diameter) to make plates. 

Inhibitory activities of the compounds against tested fungal pathogens were evaluated *in vitro* using the mycelial growth rate methods which given in [37]. Carbendazole was used as positive control and 1% acetone or DMSO with sterile distilled water as negative control. Three replicates were performed for each experiment. The inhibition rate was calculated according to Equation (1):

I = (D_1_ − D_0_)/D_1_ × 100% (1)

where I is the inhibition rate, D_1_ is the average diameter of mycelia in the blank test, and D_0_ is the average diameter of mycelia in the presence of compounds. The results are given in Table 2.

#### 3.3.3. Statistical Analysis

All experimental data were calculated and analyzed using SPSS 20.0 for Windows (SPSS China, Shanghai, China).

## 4. Conclusions

A series of novel tetrasubstituted pyrazole derivatives **1**–**32** were designed and synthesized in this paper. Most of them possessed excellent activities against *B. cinerea*, *R. solani*, *V. mali*, *T. cucumeris*, *F. oxysporum*, and *F. graminearum*. Especially, compound **26** showed the highest antifungal activity against all tested fungal pathogens with EC_50_ values of 1.638–6.043 μg/mL. It was worthy of mention that the introduction of isothiocyanate and amide moieties into the pyrazole ring could dramatically enhance the activities of the target compounds. And compounds with those functional groups were worthy of further study.

## Supplementary Materials

Supplementary materials can be accessed at: http://www.mdpi.com/1420-3049/19/9/14036/s1.

## Supplementary Files

Supplementary File 1
