# Supplementary material for: Design, Synthesis and Fungicidal Activities of Some Novel Pyrazole Derivatives
Source: Molecules. 2014 Sep 8;19(9):14036–51. doi: 10.3390/molecules190914036 (PMC6271163; doi:10.3390/molecules190914036)
Supplement: Supplementary File 1 [file molecules-19-14036-s001.pdf]

Supplementary Materials

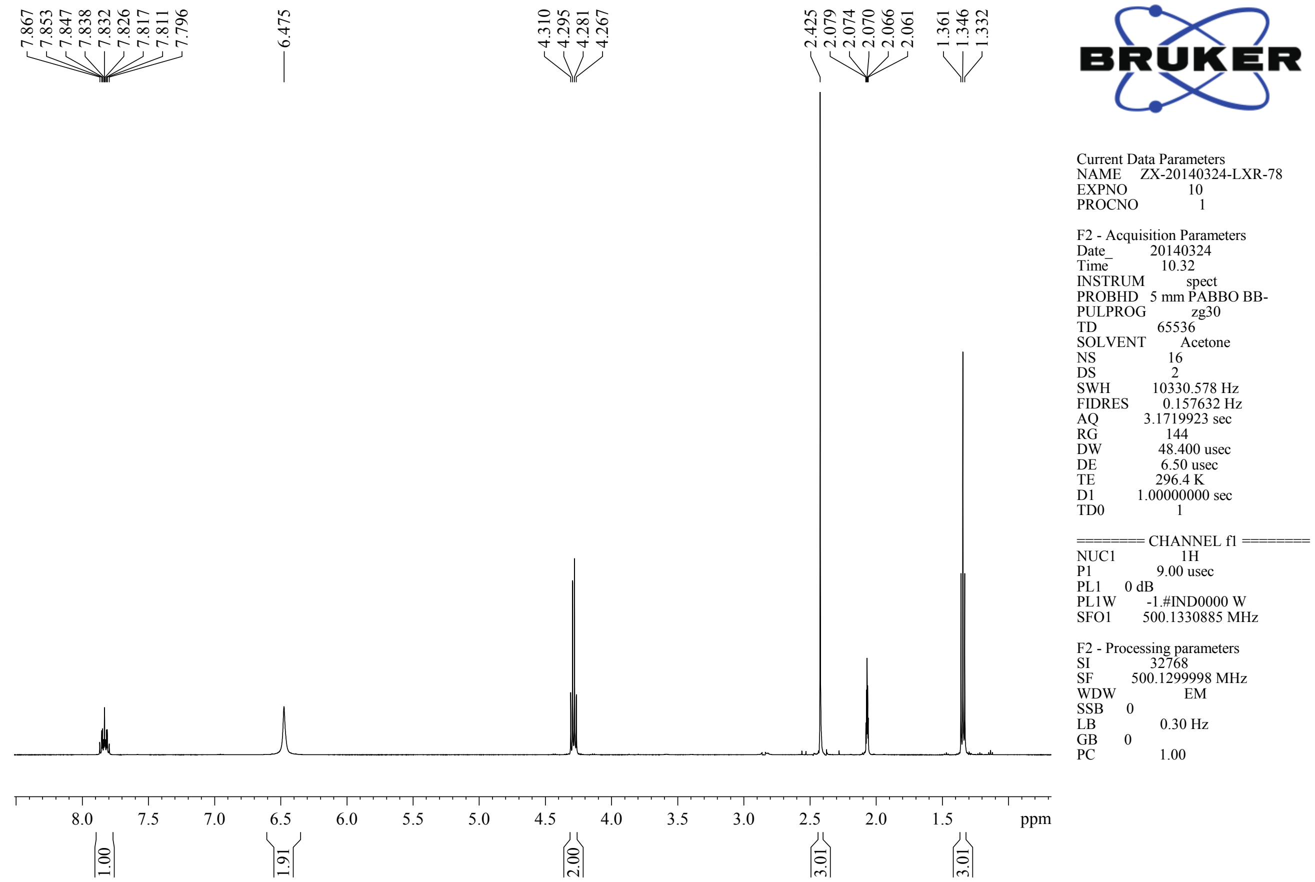

Compound **01** ^{1}H-NMR

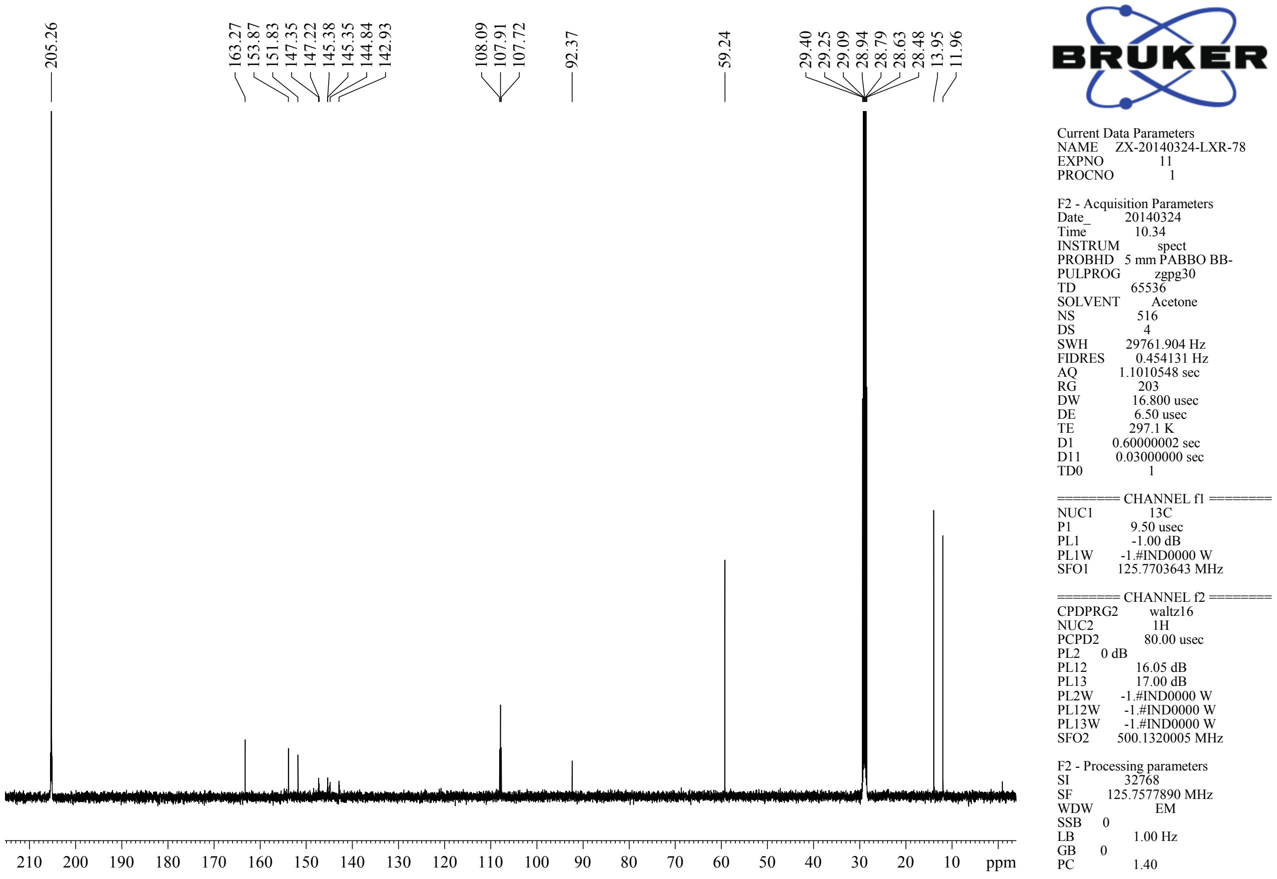

Compound **01** ^{13}C-NMR

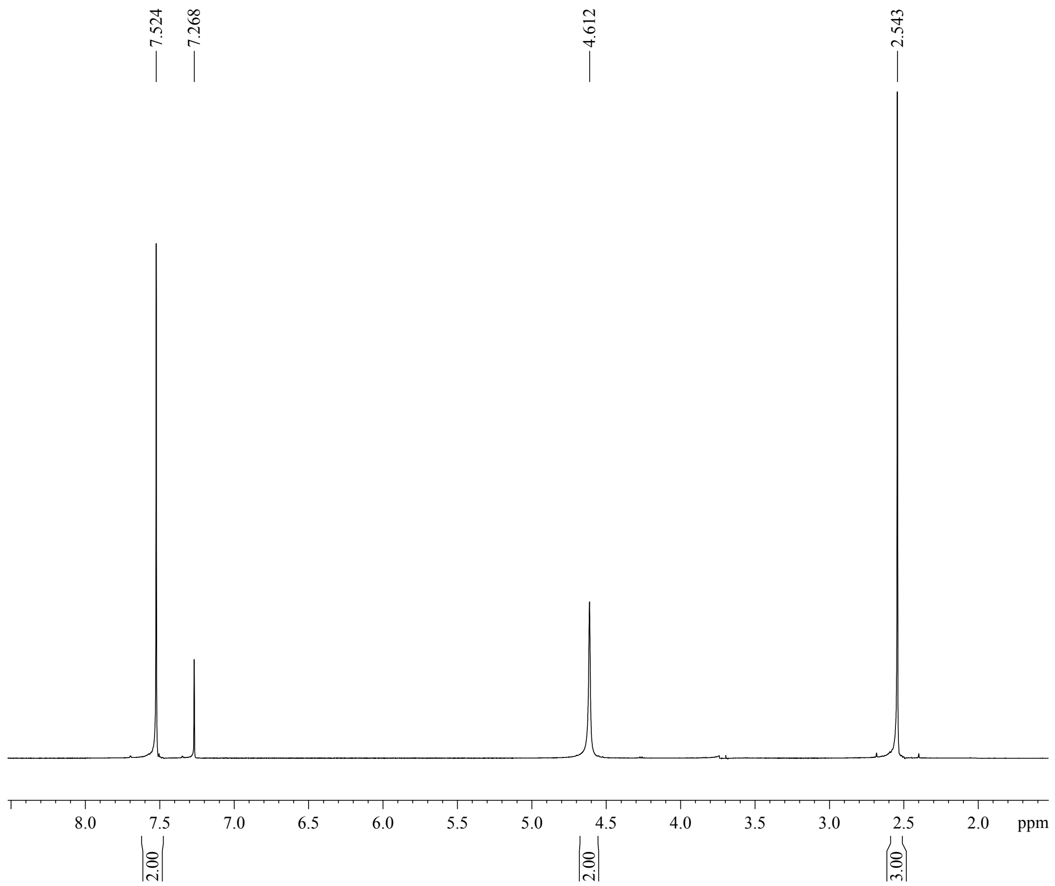

Current Data Parameters
NAME ZX-20140617-LXR-006
EXPNO 10
PROCNO 1

F2 - Acquisition Parameters
Date_ 20140617
Time 16.48
INSTRUM spect
PROBHD 5 mm PABBO BB-
PULPROG zg30
TD 65536
SOLVENT CDCl3
NS 10
DS 2
SWH 10330.578 Hz
FIDRES 0.157632 Hz
AQ 3.1719923 sec
RG 144
DW 48.400 usec
DE 6.50 usec
TE 300.1 K
D1 1.00000000 sec
TD0 1

======== CHANNEL f1 ========
NUC1 1H
P1 9.00 usec
PL1 0 dB
PL1W -1.#IND0000 W
SFO1 500.1330885 MHz

F2 - Processing parameters
SI 32768
SF 500.1300190 MHz
WDW EM
SSB 0
LB 0.30 Hz
GB 0
PC 1.00

Compound **06** ^{1}H-NMR

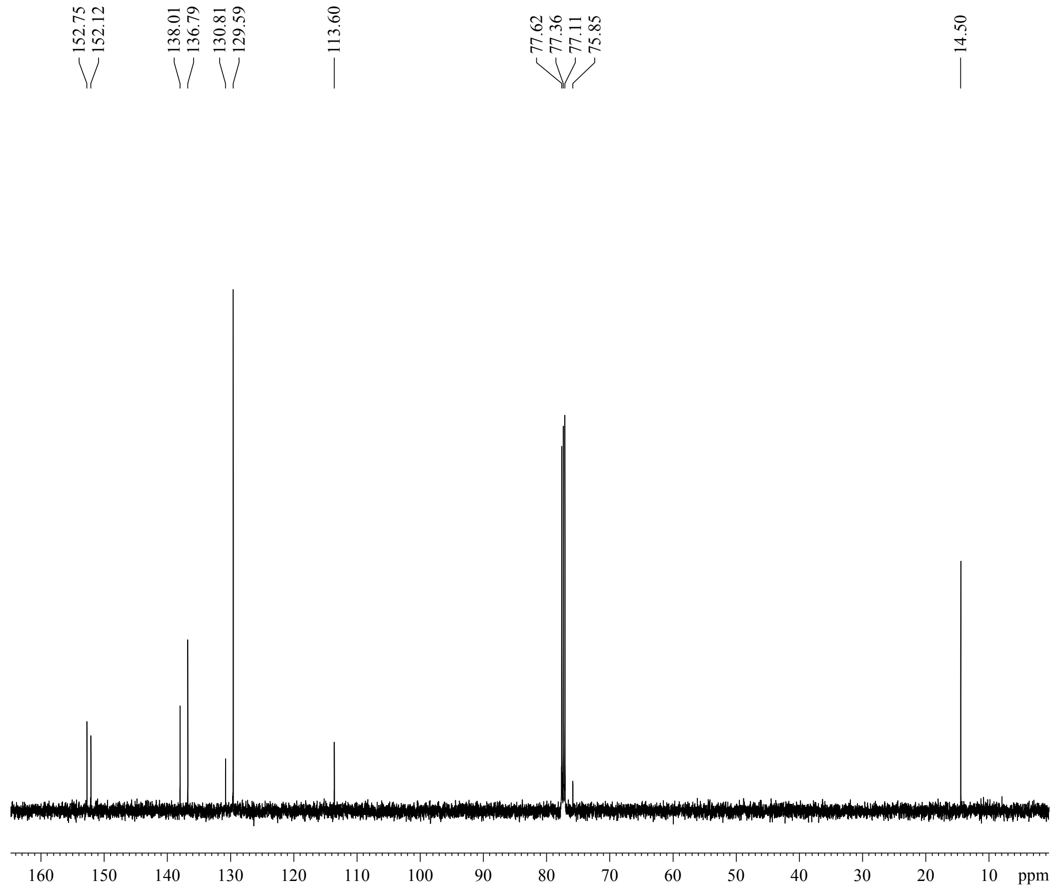

Current Data Parameters
NAME ZX-20140617-LXR-006
EXPNO 12
PROCNO 1

F2 - Acquisition Parameters
Date_ 20140617
Time 16.50
INSTRUM spect
PROBHD 5 mm PABBO BB-
PULPROG zgpg30
TD 65536
SOLVENT CDCl3
NS 85
DS 4
SWH 29761.904 Hz
FIDRES 0.454131 Hz
AQ 1.1010548 sec
RG 203
DW 16.800 usec
DE 6.50 usec
TE 300.5 K
D1 0.60000002 sec
D11 0.03000000 sec
TD0 1

======== CHANNEL f1 ========
NUC1 13C
P1 9.50 usec
PL1 -1.00 dB
PL1W -1.#IND0000 W
SFO1 125.7703643 MHz

======== CHANNEL f2 ========
CPDPRG2 waltz16
NUC2 1H
PCPD2 80.00 usec
PL2 0 dB
PL12 16.05 dB
PL13 17.00 dB
PL2W -1.#IND0000 W
PL12W -1.#IND0000 W
PL13W -1.#IND0000 W
SFO2 500.1320005 MHz

F2 - Processing parameters
SI 32768
SF 125.7577516 MHz
WDW EM
SSB 0
LB 1.00 Hz
GB 0
PC 1.40

Compound **06** ^{13}C-NMR

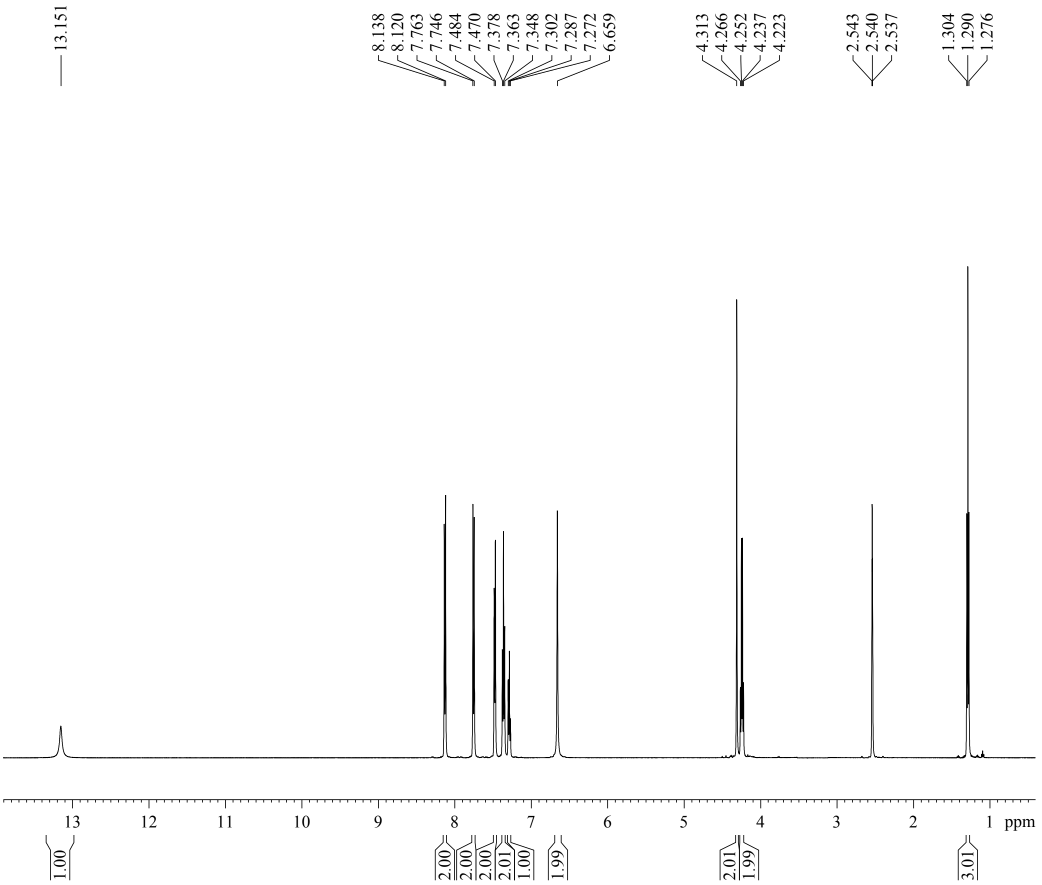

Current Data Parameters
NAME ZX-0118-LXR-55
EXPNO 10
PROCNO 1

F2 - Acquisition Parameters
Date_ 20140118
Time 13.35
INSTRUM spect
PROBHD 5 mm PABBO BB-
PULPROG zg30
TD 65536
SOLVENT DMSO
NS 16
DS 2
SWH 10330.578 Hz
FIDRES 0.157632 Hz
AQ 3.1719923 sec
RG 128
DW 48.400 usec
DE 6.50 usec
TE 297.5 K
D1 1.00000000 sec
TD0 1

======== CHANNEL f1 ========
NUC1 1H
P1 9.00 usec
PL1 0 dB
PL1W -1.#IND0000 W
SFO1 500.1330885 MHz

F2 - Processing parameters
SI 32768
SF 500.1299846 MHz
WDW EM
SSB 0
LB 0.30 Hz
GB 0
PC 1.00

Compound **10** ^{1}H-NMR

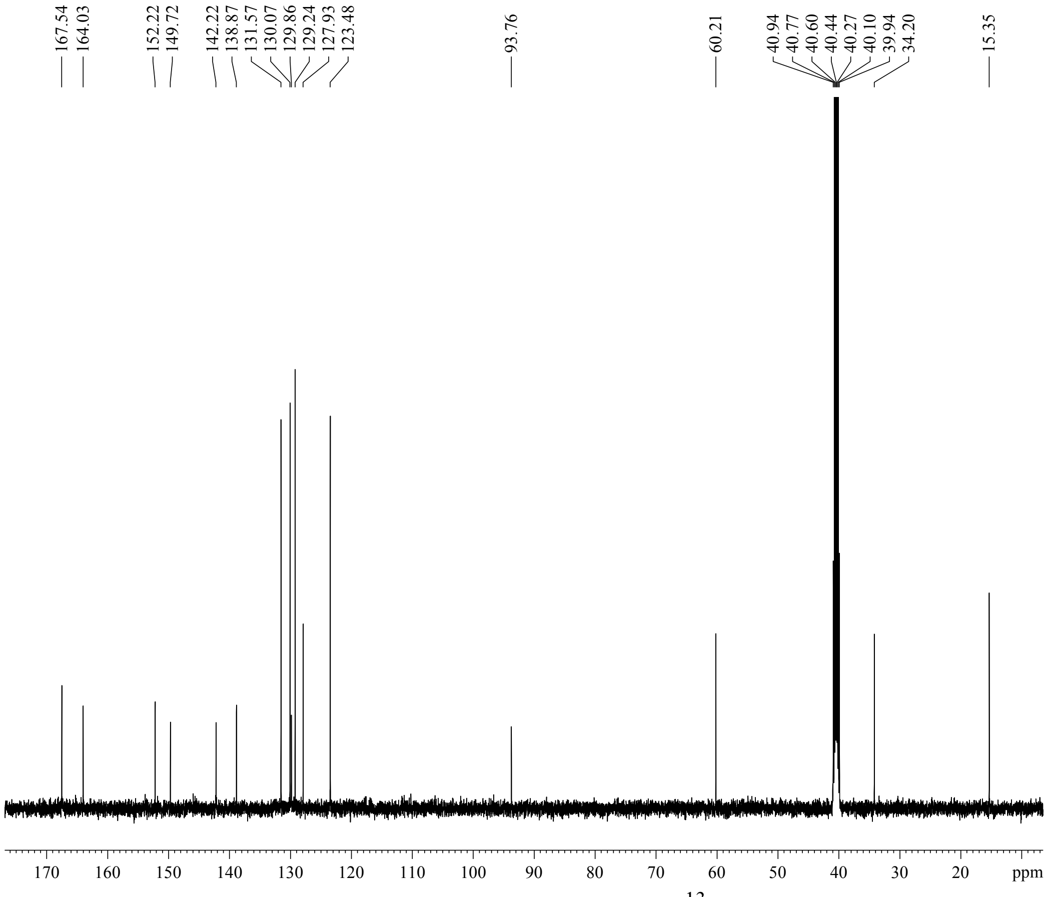

Current Data Parameters
NAME ZX-0118-LXR-55
EXPNO 11
PROCNO 1

F2 - Acquisition Parameters
Date_ 20140118
Time 13.46
INSTRUM spect
PROBHD 5 mm PABBO BB-
PULPROG zgpg30
TD 65536
SOLVENT DMSO
NS 320
DS 4
SWH 29761.904 Hz
FIDRES 0.454131 Hz
AQ 1.1010548 sec
RG 203
DW 16.800 usec
DE 6.50 usec
TE 298.5 K
D1 0.60000002 sec
D11 0.03000000 sec
TD0 1

======== CHANNEL f1 ========
NUC1 13C
P1 9.50 usec
PL1 -1.00 dB
PL1W -1.#IND0000 W
SFO1 125.7703643 MHz

======== CHANNEL f2 ========
CPDPRG2 waltz16
NUC2 1H
PCPD2 80.00 usec
PL2 0 dB
PL12 16.05 dB
PL13 17.00 dB
PL2W -1.#IND0000 W
PL12W -1.#IND0000 W
PL13W -1.#IND0000 W
SFO2 500.1320005 MHz

F2 - Processing parameters
SI 32768
SF 125.7577328 MHz
WDW EM
SSB 0
LB 1.00 Hz
GB 0
PC 1.40

Compound **10** ^{13}C-NMR

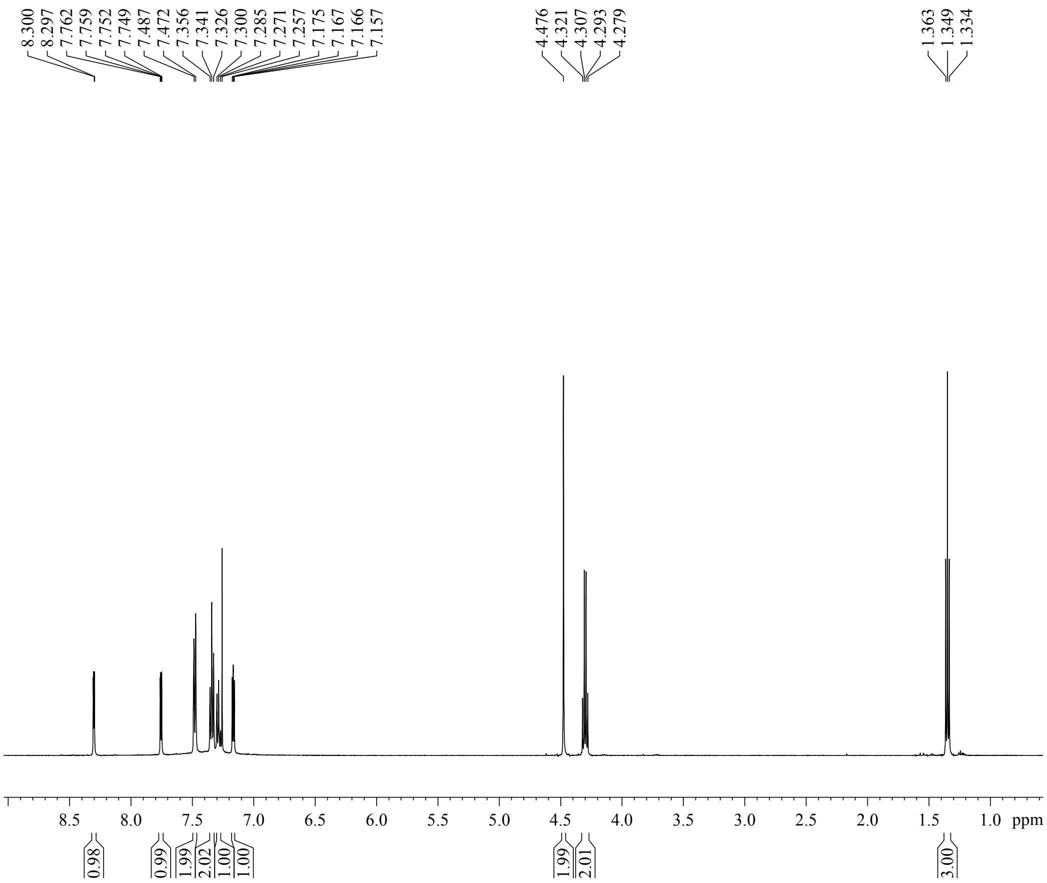

Current Data Parameters
NAME ZX-LXR-10
EXPNO 10
PROCNO 1

F2 - Acquisition Parameters
Date_ 20131025
Time 9.50
INSTRUM spect
PROBHD 5 mm PABBO BB-
PULPROG zg30
TD 65536
SOLVENT CDCl3
NS 8
DS 2
SWH 10330.578 Hz
FIDRES 0.157632 Hz
AQ 3.1719923 sec
RG 181
DW 48.400 usec
DE 6.50 usec
TE 298.7 K
D1 1.00000000 sec
TD0 1

======= CHANNEL f1 =======
NUC1 1H
P1 9.00 usec
PL1 0 dB
PL1W -1.#IND0000 W
SFO1 500.1330885 MHz

F2 - Processing parameters
SI 32768
SF 500.1300246 MHz
WDW EM
SSB 0
LB 0.30 Hz
GB 0
PC 1.00

Compound **13** ^{1}H-NMR

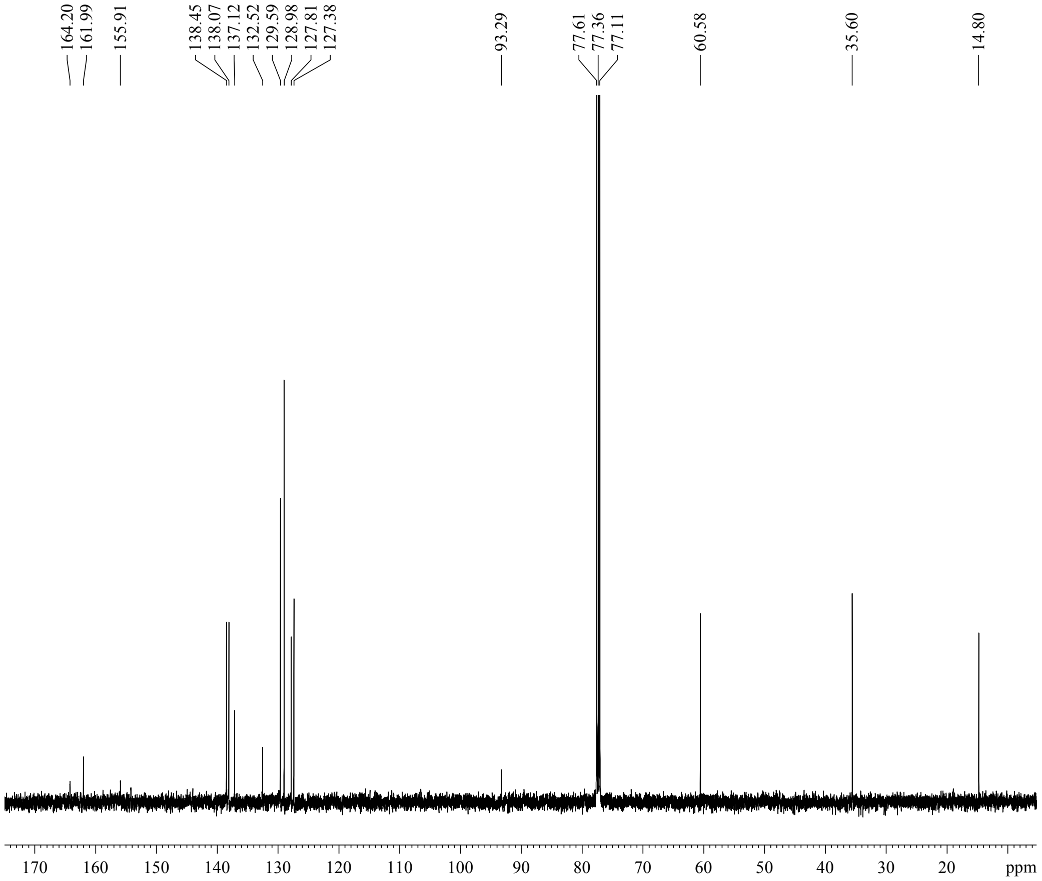

Current Data Parameters
NAME ZX-LXR-10
EXPNO 11
PROCNO 1

F2 - Acquisition Parameters
Date_ 20131025
Time 9.54
INSTRUM spect
PROBHD 5 mm PABBO BB-
PULPROG zgpg30
TD 65536
SOLVENT CDCl3
NS 360
DS 4
SWH 29761.904 Hz
FIDRES 0.454131 Hz
AQ 1.1010548 sec
RG 203
DW 16.800 usec
DE 6.50 usec
TE 299.5 K
D1 0.60000002 sec
D11 0.03000000 sec
TD0 1

======= CHANNEL f1 =======
NUC1 13C
P1 9.50 usec
PL1 -1.00 dB
PL1W -1.#IND0000 W
SFO1 125.7703643 MHz

======= CHANNEL f2 =======
CPDPRG2 waltz16
NUC2 1H
PCPD2 80.00 usec
PL2 0 dB
PL12 16.05 dB
PL13 17.00 dB
PL2W -1.#IND0000 W
PL12W -1.#IND0000 W
PL13W -1.#IND0000 W
SFO2 500.1320005 MHz

F2 - Processing parameters
SI 32768
SF 125.7577491 MHz
WDW EM
SSB 0
LB 1.00 Hz
GB 0
PC 1.40

Compound **13** ^{13}C-NMR

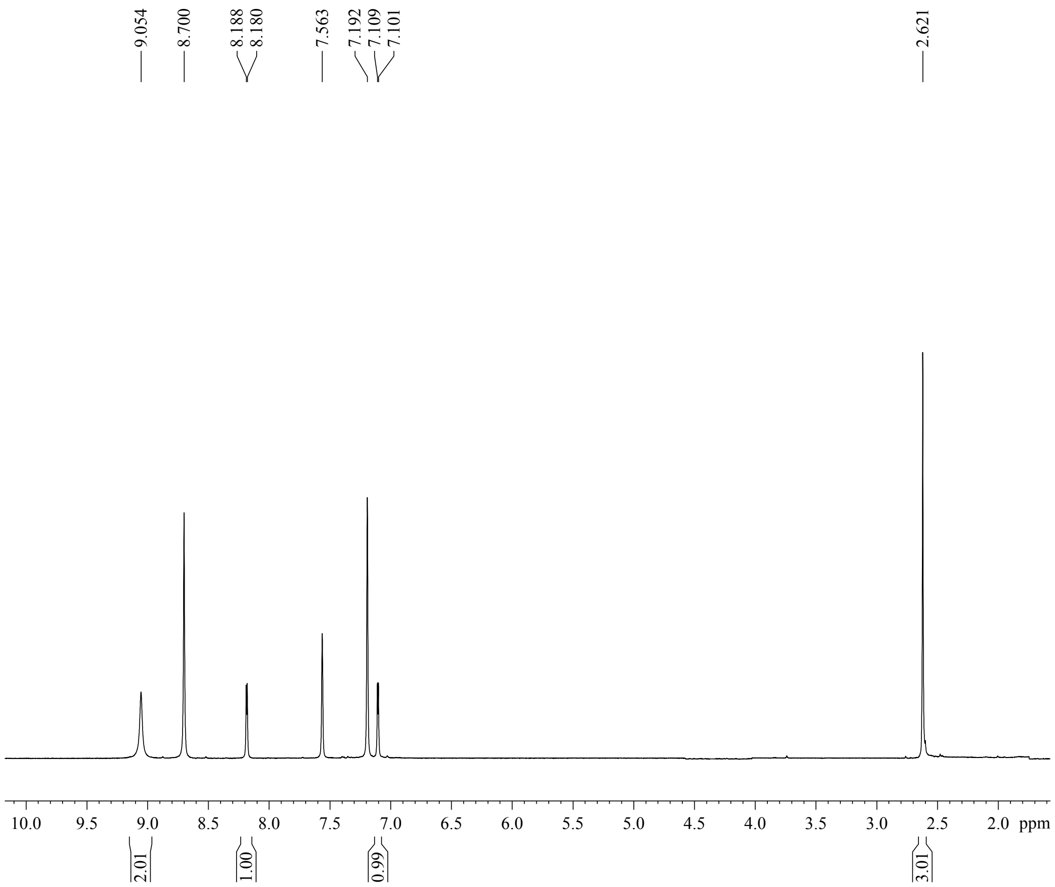

Current Data Parameters
NAME ZX-0107-LXR-45
EXPNO 10
PROCNO 1

F2 - Acquisition Parameters
Date_ 20140107
Time 17.35
INSTRUM spect
PROBHD 5 mm PABBO BB-
PULPROG zg30
TD 65536
SOLVENT Pyr
NS 8
DS 2
SWH 10330.578 Hz
FIDRES 0.157632 Hz
AQ 3.1719923 sec
RG 203
DW 48.400 usec
DE 6.50 usec
TE 332.9 K
D1 1.00000000 sec
TD0 1

======== CHANNEL f1 ========
NUC1 1H
P1 9.00 usec
PL1 0 dB
PL1W -1.#IND0000 W
SFO1 500.1330885 MHz

F2 - Processing parameters
SI 32768
SF 500.1307528 MHz
WDW EM
SSB 0
LB 0.30 Hz
GB 0
PC 1.00

Compound **15** ^{1}H-NMR

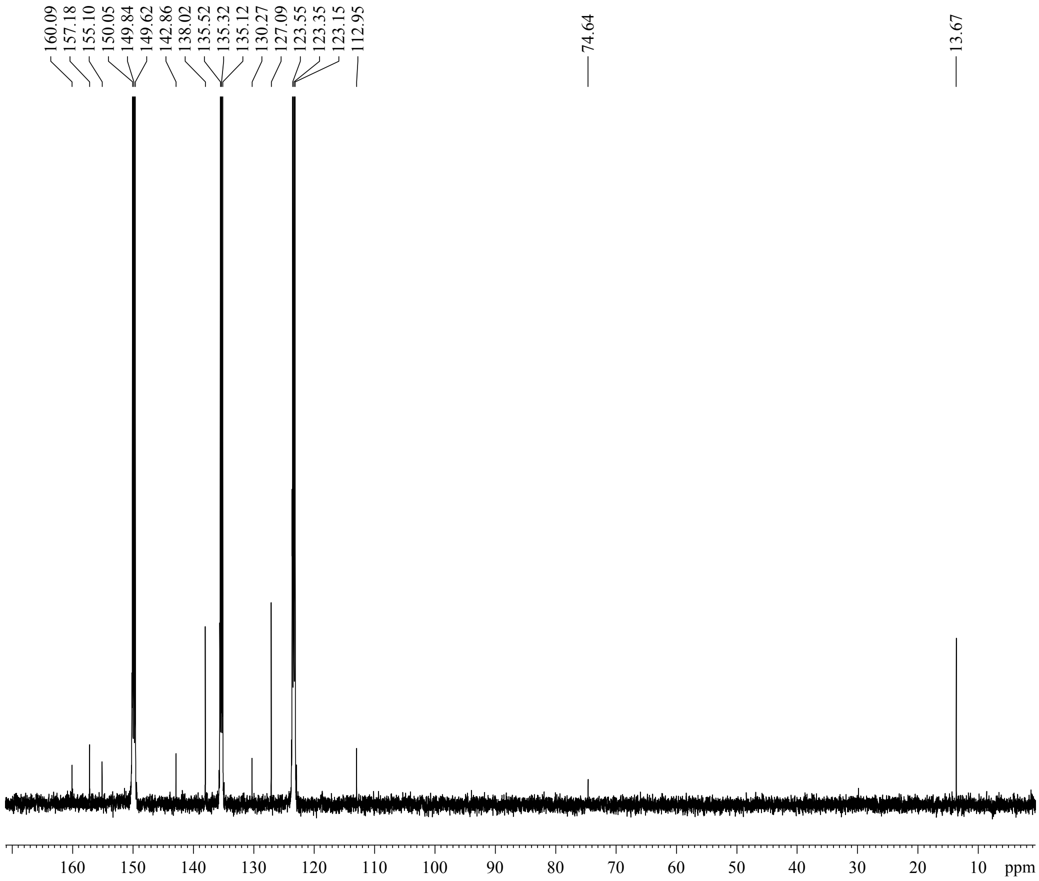

Current Data Parameters
NAME ZX-0107-LXR-45
EXPNO 11
PROCNO 1

F2 - Acquisition Parameters
Date_ 20140107
Time 17.45
INSTRUM spect
PROBHD 5 mm PABBO BB-
PULPROG zgpg30
TD 65536
SOLVENT Pyr
NS 1408
DS 4
SWH 29761.904 Hz
FIDRES 0.454131 Hz
AQ 1.1010548 sec
RG 203
DW 16.800 usec
DE 6.50 usec
TE 333.0 K
D1 0.60000002 sec
D11 0.03000000 sec
TD0 1

======== CHANNEL f1 ========
NUC1 13C
P1 9.50 usec
PL1 -1.00 dB
PL1W -1.#IND0000 W
SFO1 125.7703643 MHz

======== CHANNEL f2 ========
CPDPRG2 waltz16
NUC2 1H
PCPD2 80.00 usec
PL2 0 dB
PL12 16.05 dB
PL13 17.00 dB
PL2W -1.#IND0000 W
PL12W -1.#IND0000 W
PL13W -1.#IND0000 W
SFO2 500.1320005 MHz

F2 - Processing parameters
SI 32768
SF 125.7579527 MHz
WDW EM
SSB 0
LB 1.00 Hz
GB 0
PC 1.40

Compound **15** ^{13}C-NMR

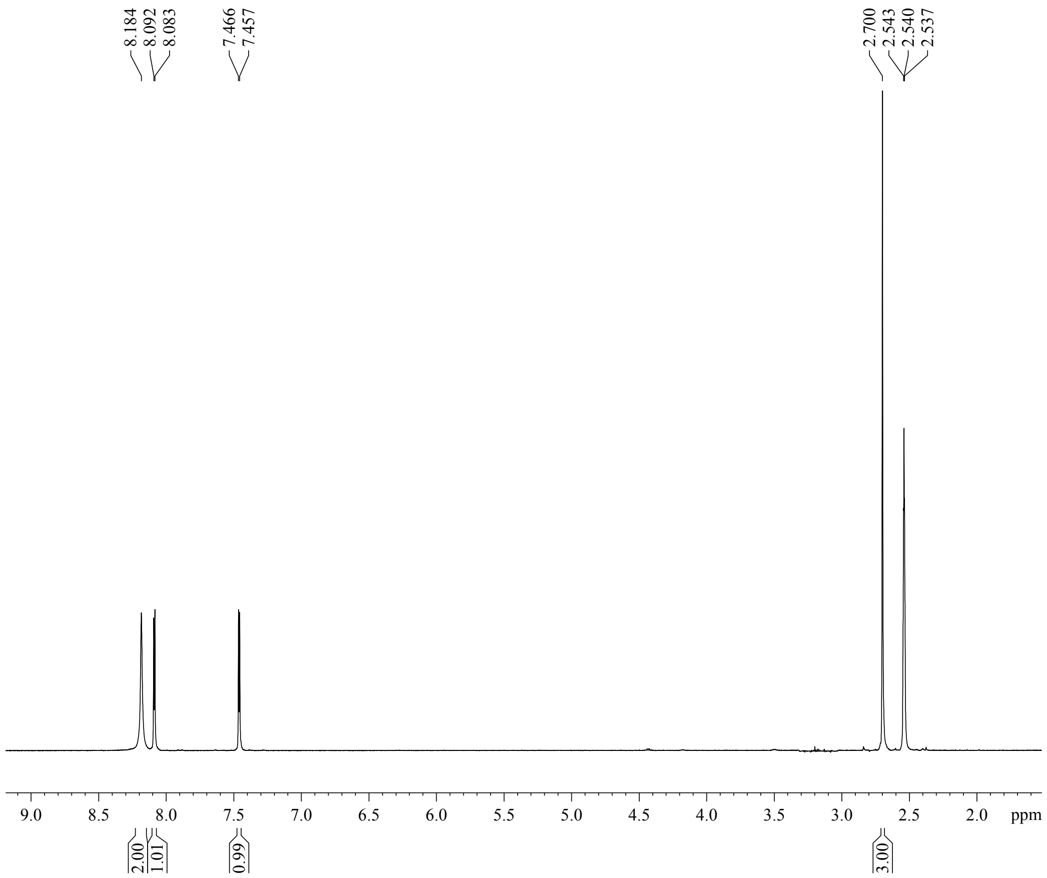

Current Data Parameters
NAME ZX-0107-LXR-44
EXPNO 10
PROCNO 1

F2 - Acquisition Parameters
Date_ 20140107
Time 17.09
INSTRUM spect
PROBHD 5 mm PABBO BB-
PULPROG zg30
TD 65536
SOLVENT DMSO
NS 8
DS 2
SWH 10330.578 Hz
FIDRES 0.157632 Hz
AQ 3.1719923 sec
RG 203
DW 48.400 usec
DE 6.50 usec
TE 333.8 K
D1 1.00000000 sec
TD0 1

======== CHANNEL f1 ========
NUC1 1H
P1 9.00 usec
PL1 0 dB
PL1W -1.#IND0000 W
SFO1 500.1330885 MHz

F2 - Processing parameters
SI 32768
SF 500.1299851 MHz
WDW EM
SSB 0
LB 0.30 Hz
GB 0
PC 1.00

Compound **18** ^{1}H-NMR

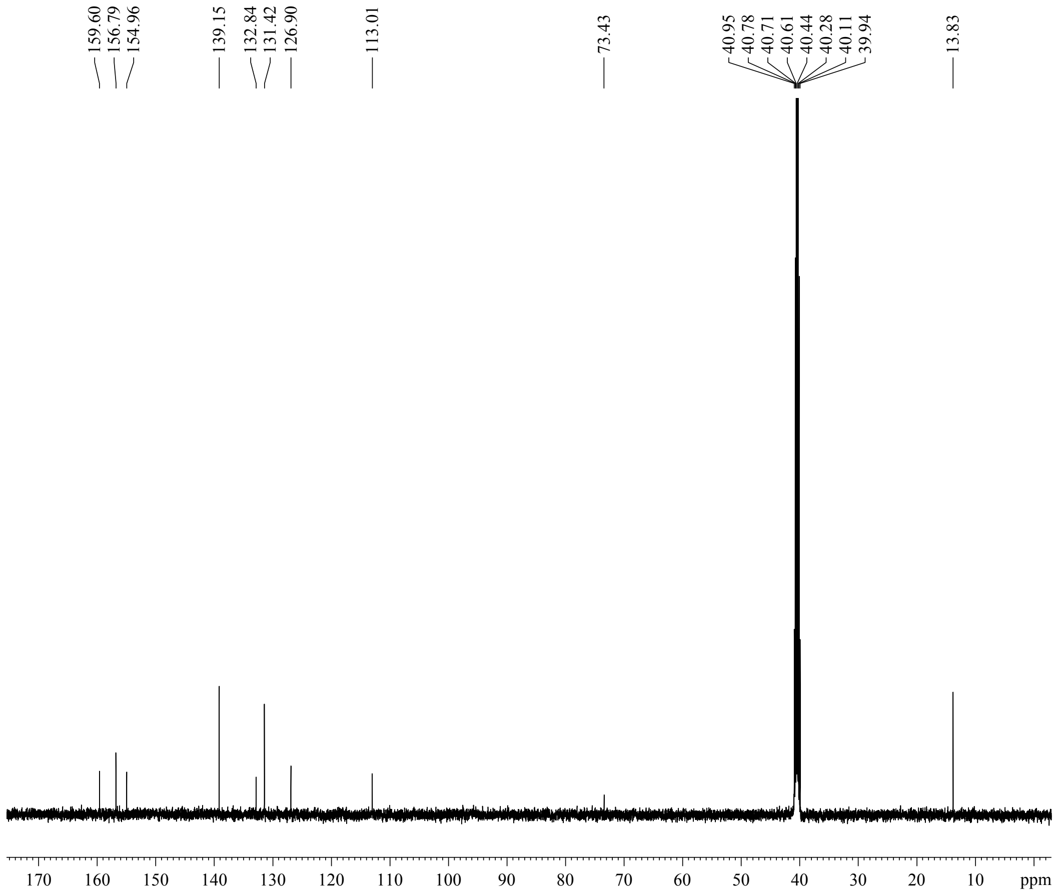

Current Data Parameters
NAME ZX-0107-LXR-44
EXPNO 11
PROCNO 1

F2 - Acquisition Parameters
Date_ 20140107
Time 17.11
INSTRUM spect
PROBHD 5 mm PABBO BB-
PULPROG zgpg30
TD 65536
SOLVENT DMSO
NS 649
DS 4
SWH 29761.904 Hz
FIDRES 0.454131 Hz
AQ 1.1010548 sec
RG 203
DW 16.800 usec
DE 6.50 usec
TE 333.4 K
D1 0.60000002 sec
D11 0.03000000 sec
TD0 1

======== CHANNEL f1 ========
NUC1 13C
P1 9.50 usec
PL1 -1.00 dB
PL1W -1.#IND0000 W
SFO1 125.7703643 MHz

======== CHANNEL f2 ========
CPDPRG2 waltz16
NUC2 1H
PCPD2 80.00 usec
PL2 0 dB
PL12 16.05 dB
PL13 17.00 dB
PL2W -1.#IND0000 W
PL12W -1.#IND0000 W
PL13W -1.#IND0000 W
SFO2 500.1320005 MHz

F2 - Processing parameters
SI 32768
SF 125.7577688 MHz
WDW EM
SSB 0
LB 1.00 Hz
GB 0
PC 1.40

Compound **18** ^{13}C-NMR

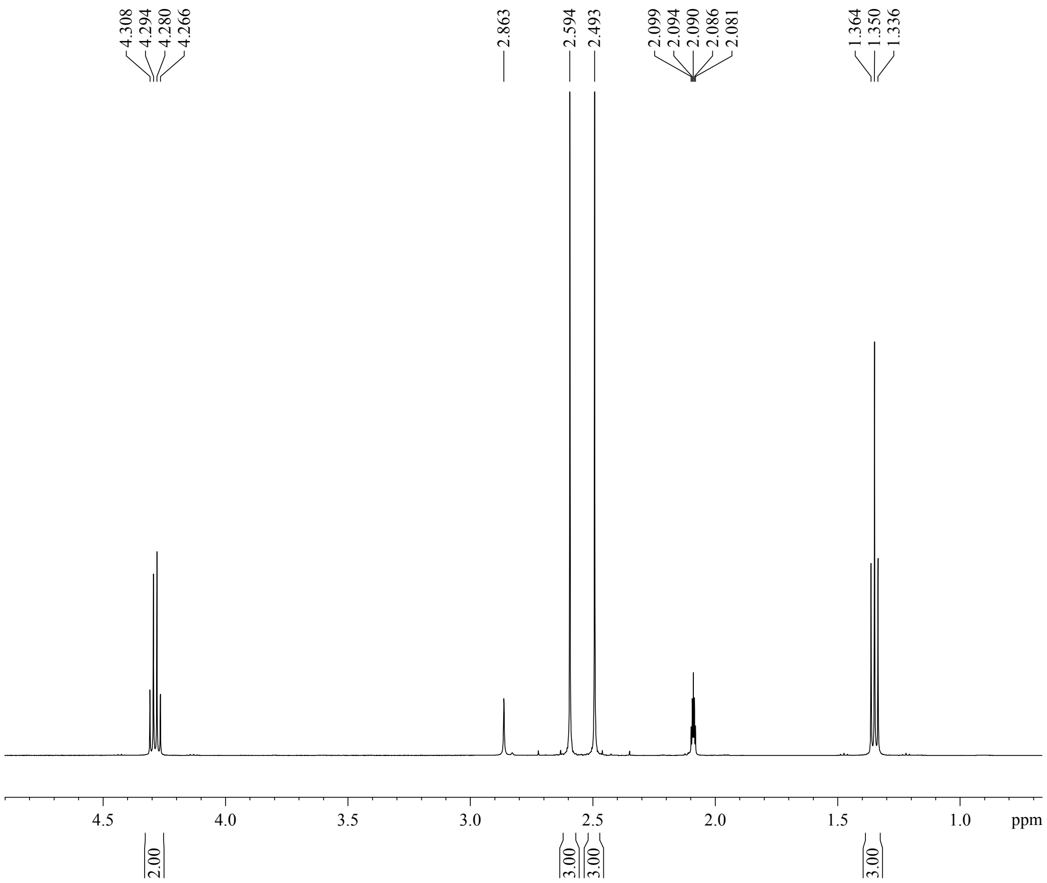

Current Data Parameters
NAME ZX-20140324-LXR-79
EXPNO 10
PROCNO 1

F2 - Acquisition Parameters
Date_ 20140324
Time 10.53
INSTRUM spect
PROBHD 5 mm PABBO BB-
PULPROG zg30
TD 65536
SOLVENT Acetone
NS 16
DS 2
SWH 10330.578 Hz
FIDRES 0.157632 Hz
AQ 3.1719923 sec
RG 114
DW 48.400 usec
DE 6.50 usec
TE 296.4 K
D1 1.00000000 sec
TD0 1

======== CHANNEL f1 ========
NUC1 1H
P1 9.00 usec
PL1 0 dB
PL1W -1.#IND0000 W
SFO1 500.1330885 MHz

F2 - Processing parameters
SI 32768
SF 500.1299898 MHz
WDW EM
SSB 0
LB 0.30 Hz
GB 0
PC 1.00

Compound **19** ^{1}H-NMR

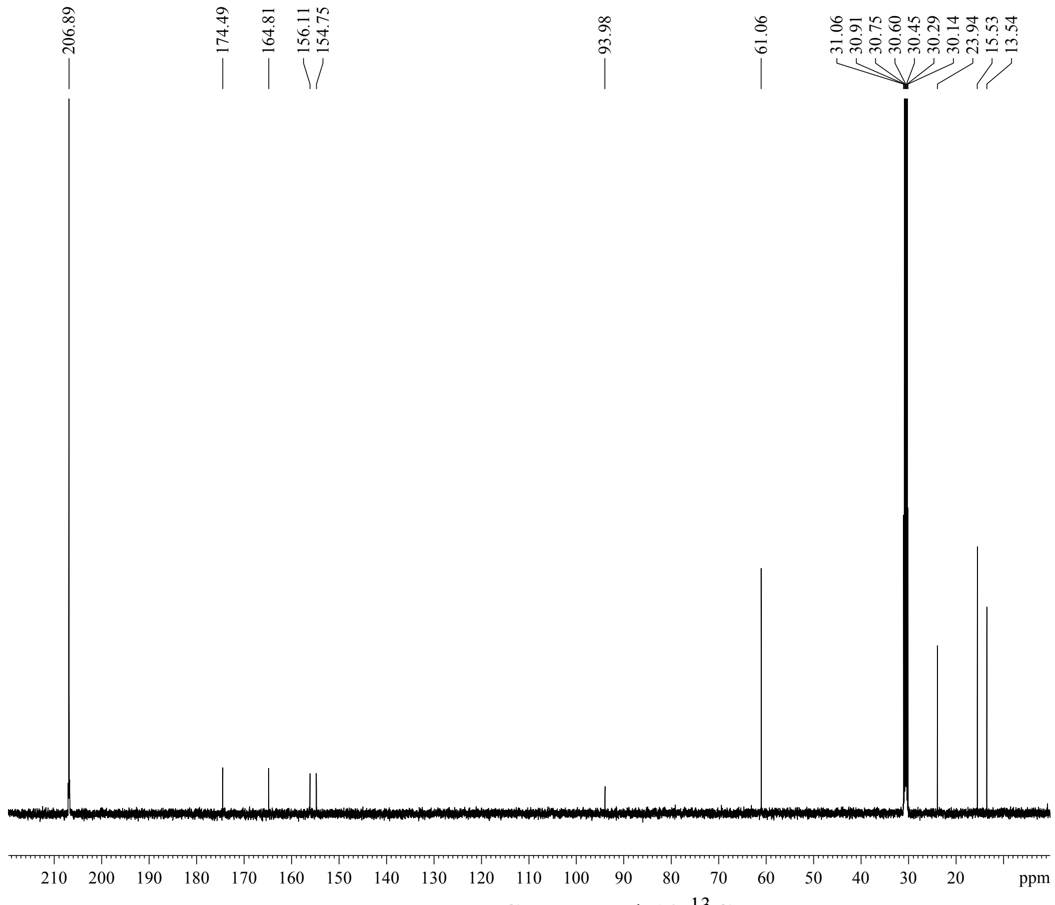

Current Data Parameters
NAME ZX-20140324-LXR-79
EXPNO 11
PROCNO 1

F2 - Acquisition Parameters
Date_ 20140324
Time 10.54
INSTRUM spect
PROBHD 5 mm PABBO BB-
PULPROG zgpg30
TD 65536
SOLVENT Acetone
NS 403
DS 4
SWH 29761.904 Hz
FIDRES 0.454131 Hz
AQ 1.1010548 sec
RG 203
DW 16.800 usec
DE 6.50 usec
TE 296.8 K
D1 0.60000002 sec
D11 0.03000000 sec
TD0 1

======== CHANNEL f1 ========
NUC1 13C
P1 9.50 usec
PL1 -1.00 dB
PL1W -1.#IND0000 W
SFO1 125.7703643 MHz

======== CHANNEL f2 ========
CPDPRG2 waltz16
NUC2 1H
PCPD2 80.00 usec
PL2 0 dB
PL12 16.05 dB
PL13 17.00 dB
PL2W -1.#IND0000 W
PL12W -1.#IND0000 W
PL13W -1.#IND0000 W
SFO2 500.1320005 MHz

F2 - Processing parameters
SI 32768
SF 125.7575810 MHz
WDW EM
SSB 0
LB 1.00 Hz
GB 0
PC 1.40

Compound **19** ^{13}C-NMR

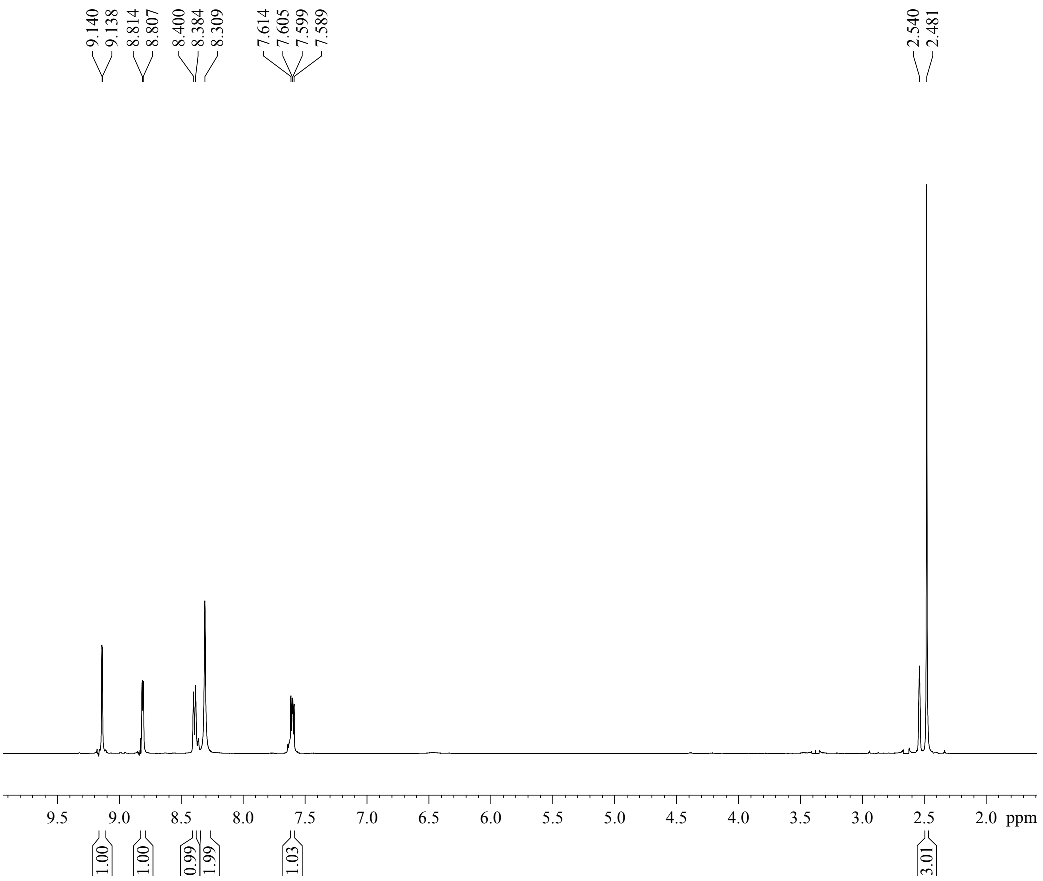

Current Data Parameters
NAME ZX-20131209-LXR-17
EXPNO 10
PROCNO 1

F2 - Acquisition Parameters
Date_ 20131209
Time 16.49
INSTRUM spect
PROBHD 5 mm PABBO BB-
PULPROG zg30
TD 65536
SOLVENT DMSO
NS 12
DS 2
SWH 10330.578 Hz
FIDRES 0.157632 Hz
AQ 3.1719923 sec
RG 114
DW 48.400 usec
DE 6.50 usec
TE 297.9 K
D1 1.00000000 sec
TD0 1

======== CHANNEL f1 ========
NUC1 1H
P1 9.00 usec
PL1 0 dB
PL1W -1.#IND0000 W
SFO1 500.1330885 MHz

F2 - Processing parameters
SI 32768
SF 500.1299847 MHz
WDW EM
SSB 0
LB 0.30 Hz
GB 0
PC 1.00

Compound **21** ^{1}H-NMR

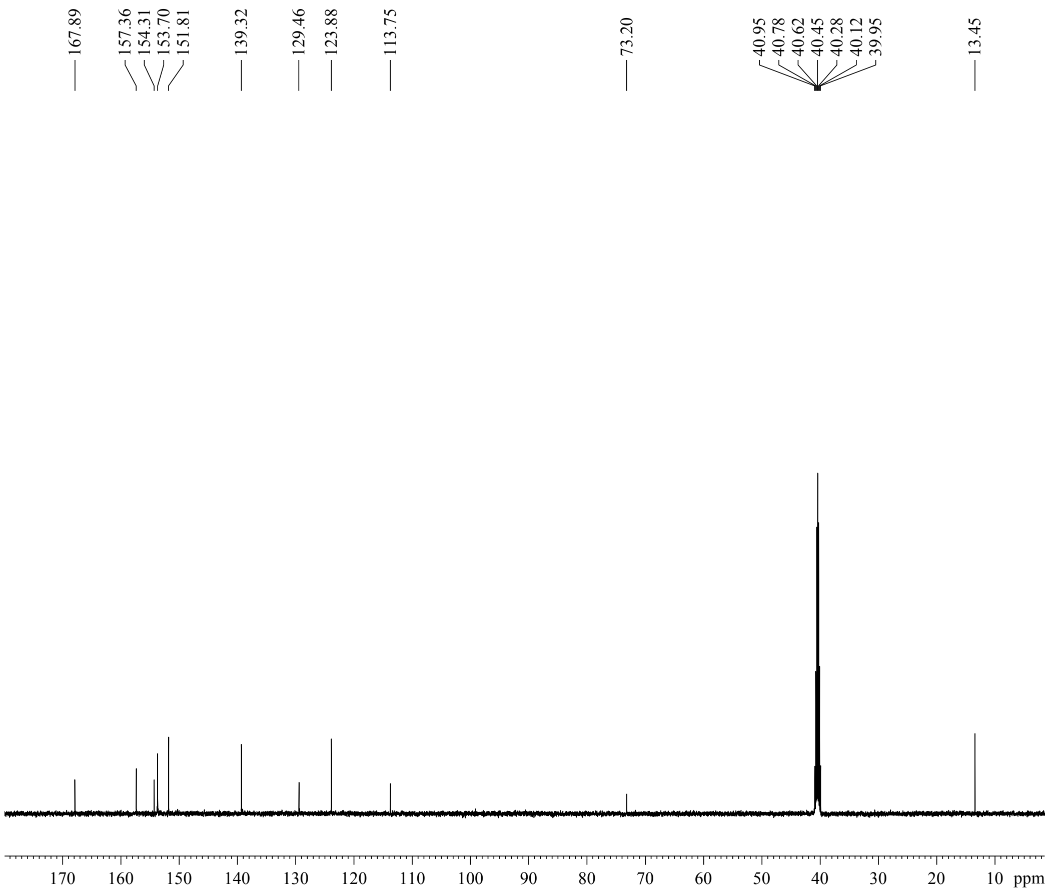

Current Data Parameters
NAME ZX-20131209-LXR-17
EXPNO 11
PROCNO 1

F2 - Acquisition Parameters
Date_ 20131209
Time 16.52
INSTRUM spect
PROBHD 5 mm PABBO BB-
PULPROG zgpg30
TD 65536
SOLVENT DMSO
NS 116
DS 4
SWH 29761.904 Hz
FIDRES 0.454131 Hz
AQ 1.1010548 sec
RG 203
DW 16.800 usec
DE 6.50 usec
TE 298.3 K
D1 0.60000002 sec
D11 0.03000000 sec
TD0 1

======== CHANNEL f1 ========
NUC1 13C
P1 9.50 usec
PL1 -1.00 dB
PL1W -1.#IND0000 W
SFO1 125.7703643 MHz

======== CHANNEL f2 ========
CPDPRG2 waltz16
NUC2 1H
PCPD2 80.00 usec
PL2 0 dB
PL12 16.05 dB
PL13 17.00 dB
PL2W -1.#IND0000 W
PL12W -1.#IND0000 W
PL13W -1.#IND0000 W
SFO2 500.1320005 MHz

F2 - Processing parameters
SI 32768
SF 125.7577305 MHz
WDW EM
SSB 0
LB 1.00 Hz
GB 0
PC 1.40

Compound **21** ^{13}C-NMR

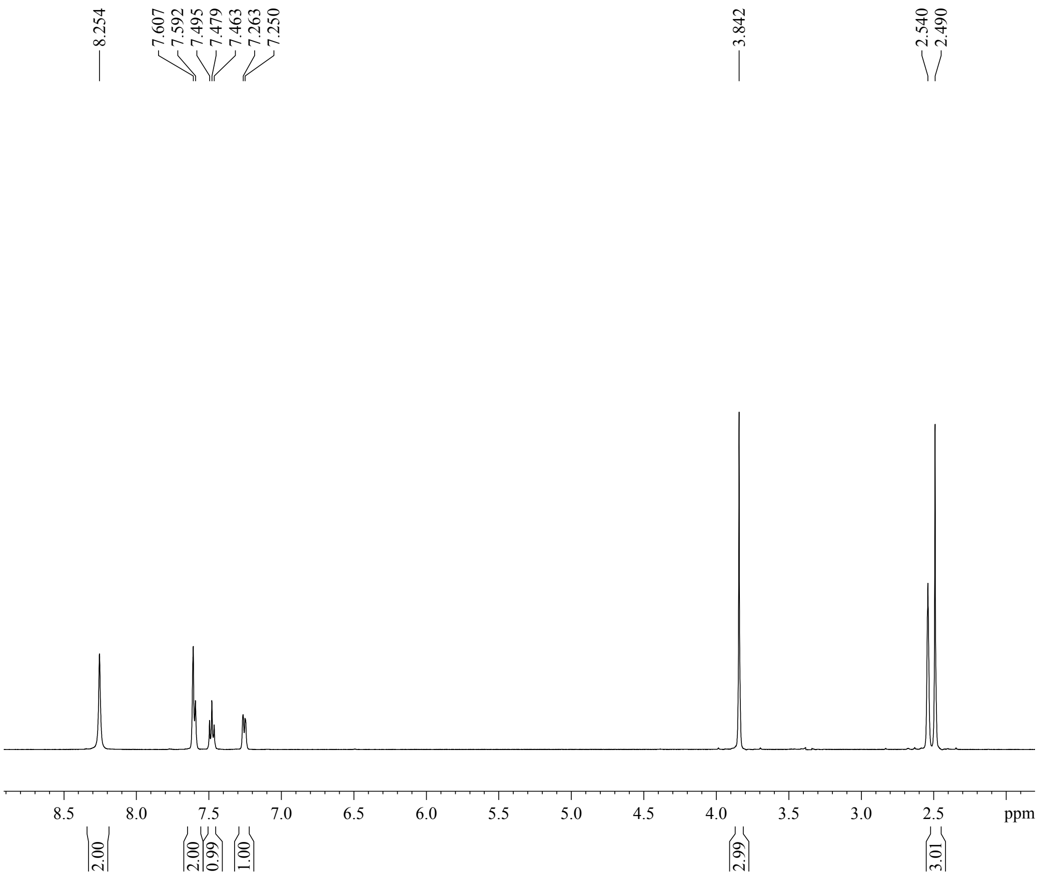

Current Data Parameters
NAME ZX-0118-LXR-51
EXPNO 10
PROCNO 1

F2 - Acquisition Parameters
Date_ 20140118
Time 12.27
INSTRUM spect
PROBHD 5 mm PABBO BB-
PULPROG zg30
TD 65536
SOLVENT DMSO
NS 15
DS 2
SWH 10330.578 Hz
FIDRES 0.157632 Hz
AQ 3.1719923 sec
RG 203
DW 48.400 usec
DE 6.50 usec
TE 298.1 K
D1 1.00000000 sec
TD0 1

======== CHANNEL f1 ========
NUC1 1H
P1 9.00 usec
PL1 0 dB
PL1W -1.#IND0000 W
SFO1 500.1330885 MHz

F2 - Processing parameters
SI 32768
SF 500.1299844 MHz
WDW EM
SSB 0
LB 0.30 Hz
GB 0
PC 1.00

Compound **25** ^{1}H-NMR

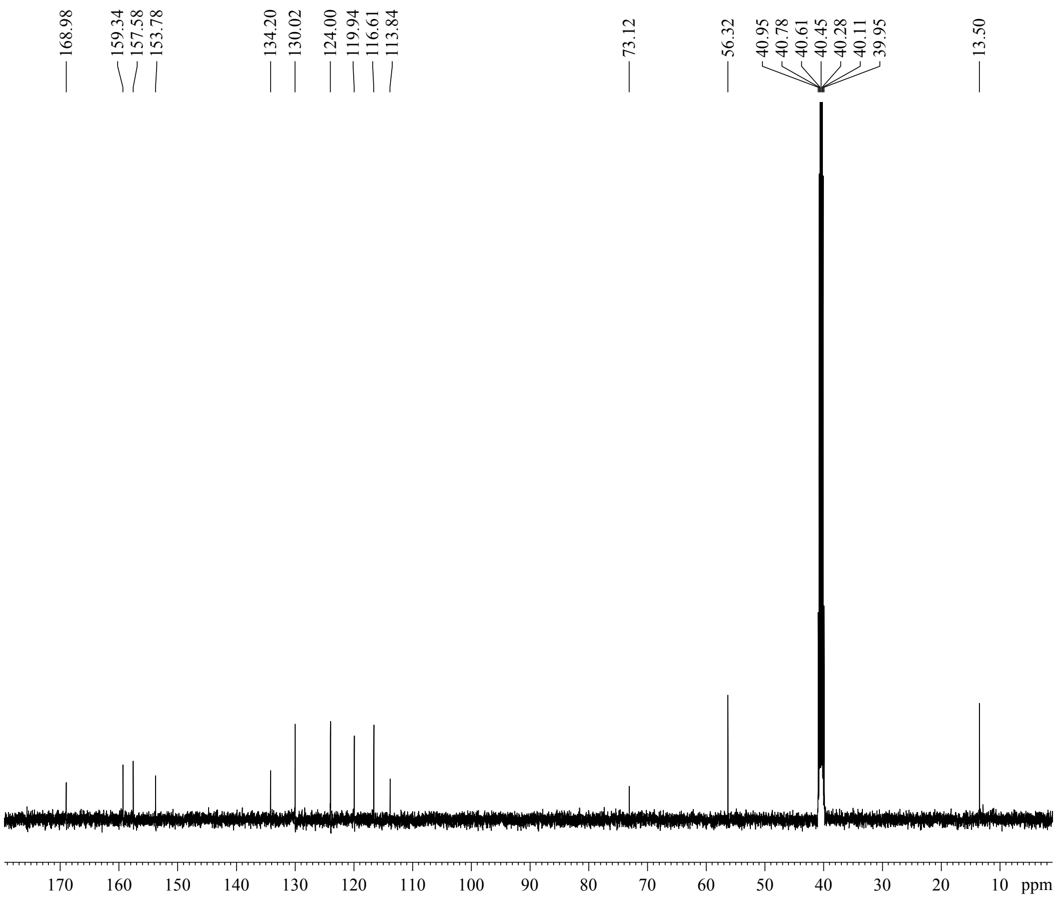

Current Data Parameters
NAME ZX-0118-LXR-51
EXPNO 11
PROCNO 1

F2 - Acquisition Parameters
Date_ 20140118
Time 12.29
INSTRUM spect
PROBHD 5 mm PABBO BB-
PULPROG zgpg30
TD 65536
SOLVENT DMSO
NS 256
DS 4
SWH 29761.904 Hz
FIDRES 0.454131 Hz
AQ 1.1010548 sec
RG 203
DW 16.800 usec
DE 6.50 usec
TE 297.9 K
D1 0.60000002 sec
D11 0.03000000 sec
TD0 1

======== CHANNEL f1 ========
NUC1 13C
P1 9.50 usec
PL1 -1.00 dB
PL1W -1.#IND0000 W
SFO1 125.7703643 MHz

======== CHANNEL f2 ========
CPDPRG2 waltz16
NUC2 1H
PCPD2 80.00 usec
PL2 0 dB
PL12 16.05 dB
PL13 17.00 dB
PL2W -1.#IND0000 W
PL12W -1.#IND0000 W
PL13W -1.#IND0000 W
SFO2 500.1320005 MHz

F2 - Processing parameters
SI 32768
SF 125.7577313 MHz
WDW EM
SSB 0
LB 1.00 Hz
GB 0
PC 1.40

Compound **25** ^{13}C-NMR

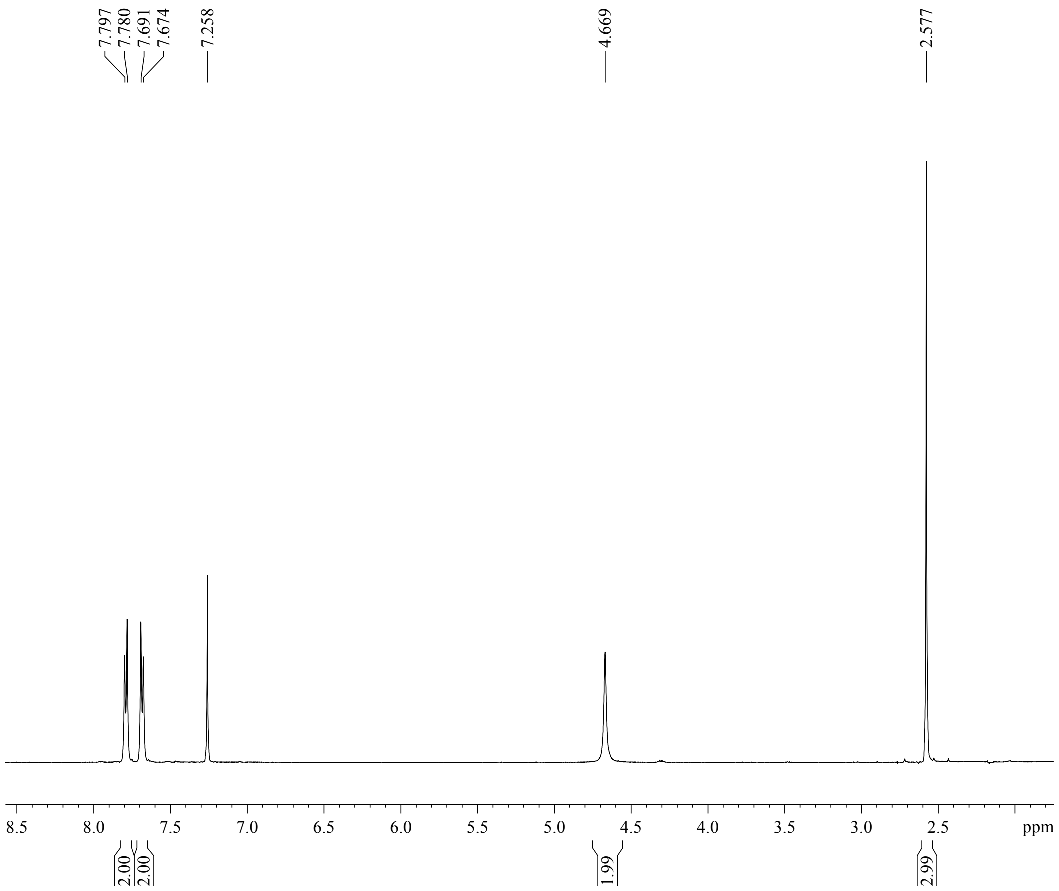

Current Data Parameters
NAME ZX-0118-LXR-48
EXPNO 10
PROCNO 1

F2 - Acquisition Parameters
Date_ 20140118
Time 10.39
INSTRUM spect
PROBHD 5 mm PABBO BB-
PULPROG zg30
TD 65536
SOLVENT CDCl3
NS 16
DS 2
SWH 10330.578 Hz
FIDRES 0.157632 Hz
AQ 3.1719923 sec
RG 161
DW 48.400 usec
DE 6.50 usec
TE 294.4 K
D1 1.00000000 sec
TD0 1

======== CHANNEL f1 ========
NUC1 1H
P1 9.00 usec
PL1 0 dB
PL1W -1.#IND0000 W
SFO1 500.1330885 MHz

F2 - Processing parameters
SI 32768
SF 500.1300239 MHz
WDW EM
SSB 0
LB 0.30 Hz
GB 0
PC 1.00

Compound **26** ^{1}H-NMR

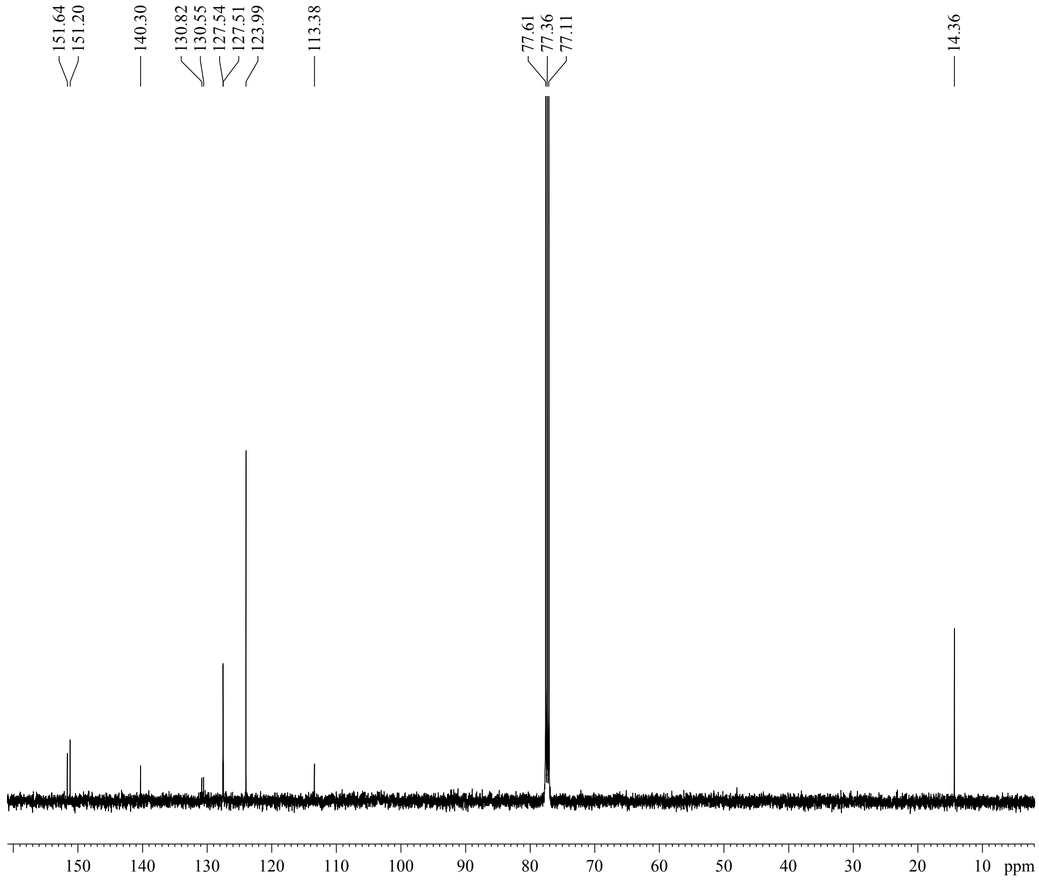

Current Data Parameters
NAME ZX-0118-LXR-48
EXPNO 11
PROCNO 1

F2 - Acquisition Parameters
Date_ 20140118
Time 10.41
INSTRUM spect
PROBHD 5 mm PABBO BB-
PULPROG zgpg30
TD 65536
SOLVENT CDCl3
NS 1063
DS 4
SWH 29761.904 Hz
FIDRES 0.454131 Hz
AQ 1.1010548 sec
RG 203
DW 16.800 usec
DE 6.50 usec
TE 295.2 K
D1 0.60000002 sec
D11 0.03000000 sec
TD0 1

======== CHANNEL f1 ========
NUC1 13C
P1 9.50 usec
PL1 -1.00 dB
PL1W -1.#IND0000 W
SFO1 125.7703643 MHz

======== CHANNEL f2 ========
CPDPRG2 waltz16
NUC2 1H
PCPD2 80.00 usec
PL2 0 dB
PL12 16.05 dB
PL13 17.00 dB
PL2W -1.#IND0000 W
PL12W -1.#IND0000 W
PL13W -1.#IND0000 W
SFO2 500.1320005 MHz

F2 - Processing parameters
SI 32768
SF 125.7577499 MHz
WDW EM
SSB 0
LB 1.00 Hz
GB 0
PC 1.40

Compound **26** ^{13}C-NMR

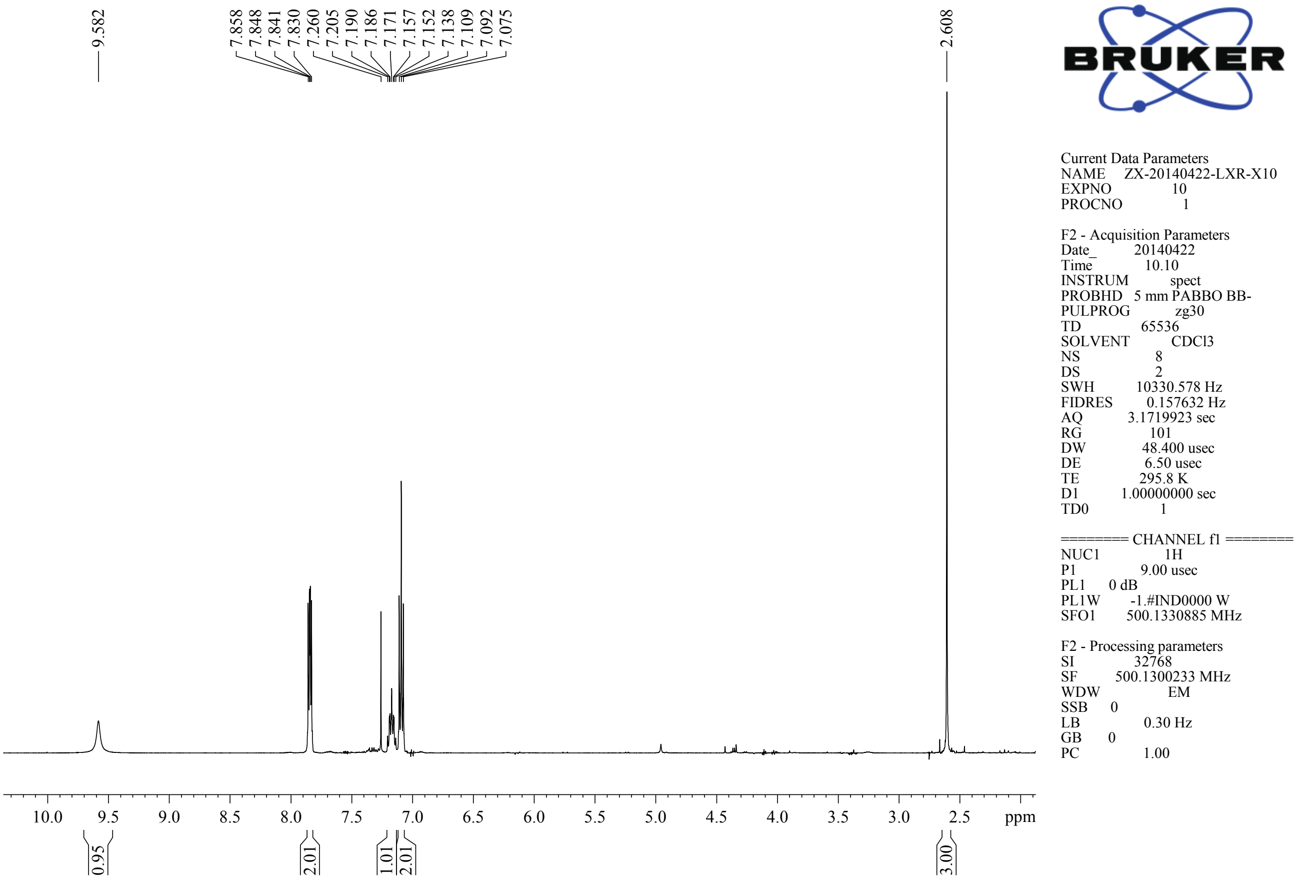

Compound **27** ^{1}H-NMR

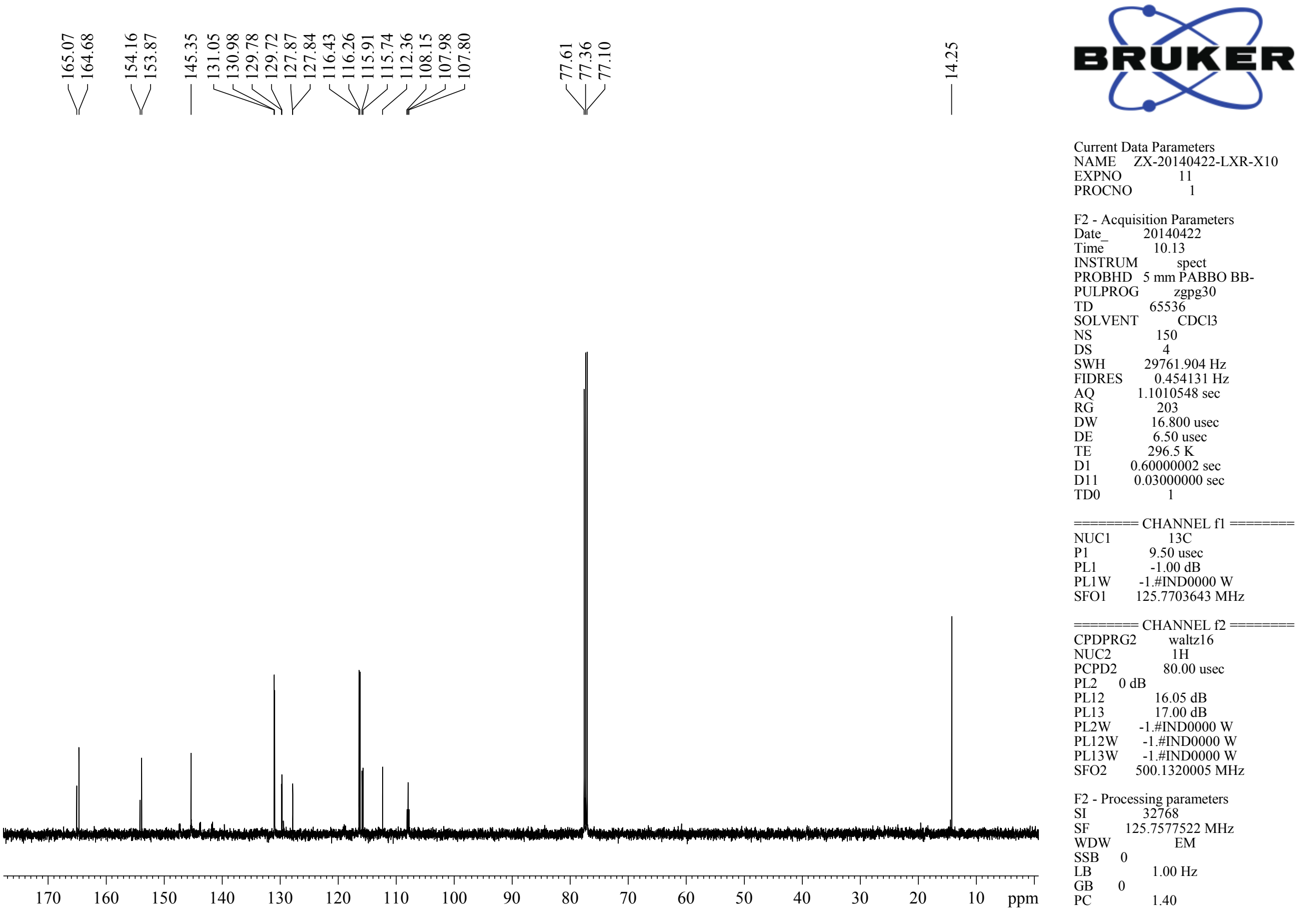

Compound **27** ^{13}C-NMR

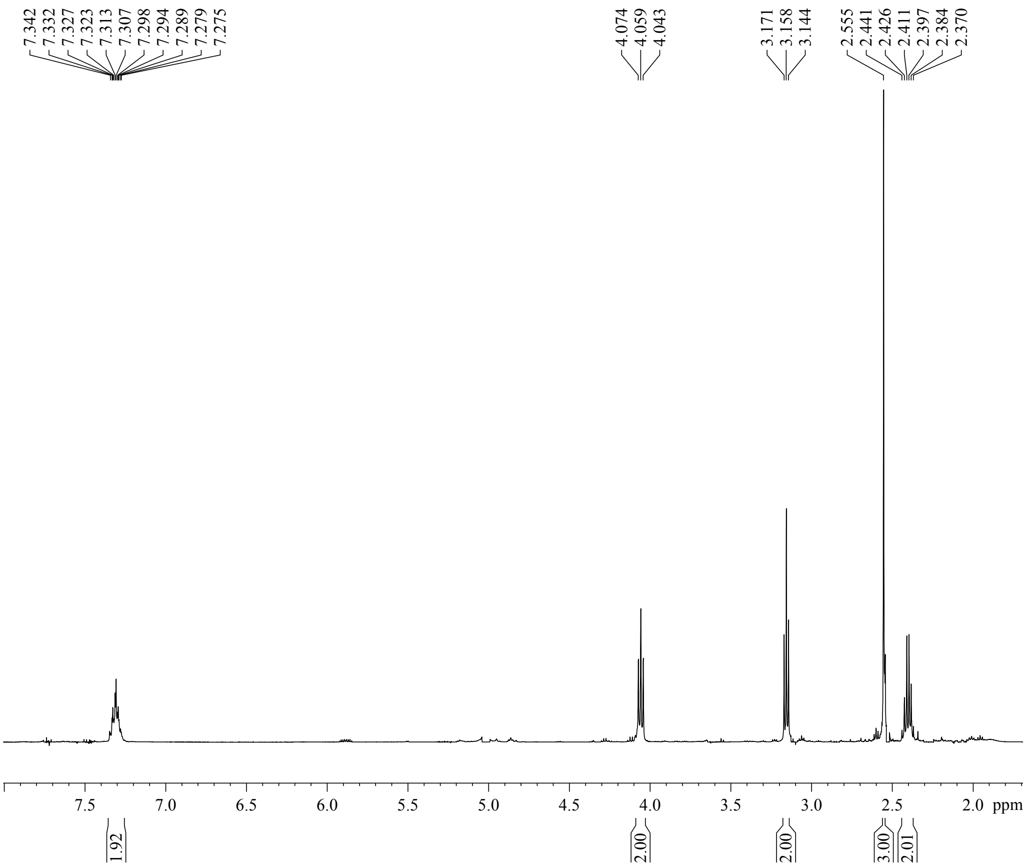

Current Data Parameters
NAME ZX-20140612-LXR-S4
EXPNO 10
PROCNO 1

F2 - Acquisition Parameters
Date_ 20140612
Time 10.17
INSTRUM spect
PROBHD 5 mm PABBO BB-
PULPROG zg30
TD 65536
SOLVENT CDCl3
NS 16
DS 2
SWH 10330.578 Hz
FIDRES 0.157632 Hz
AQ 3.1719923 sec
RG 57
DW 48.400 usec
DE 6.50 usec
TE 300.3 K
D1 1.00000000 sec
TD0 1

======== CHANNEL f1 ========
NUC1 1H
P1 9.00 usec
PL1 0 dB
PL1W -1.#IND0000 W
SFO1 500.1330885 MHz

F2 - Processing parameters
SI 32768
SF 500.1300000 MHz
WDW EM
SSB 0
LB 0.30 Hz
GB 0
PC 1.00

Compound **30** ^{1}H-NMR

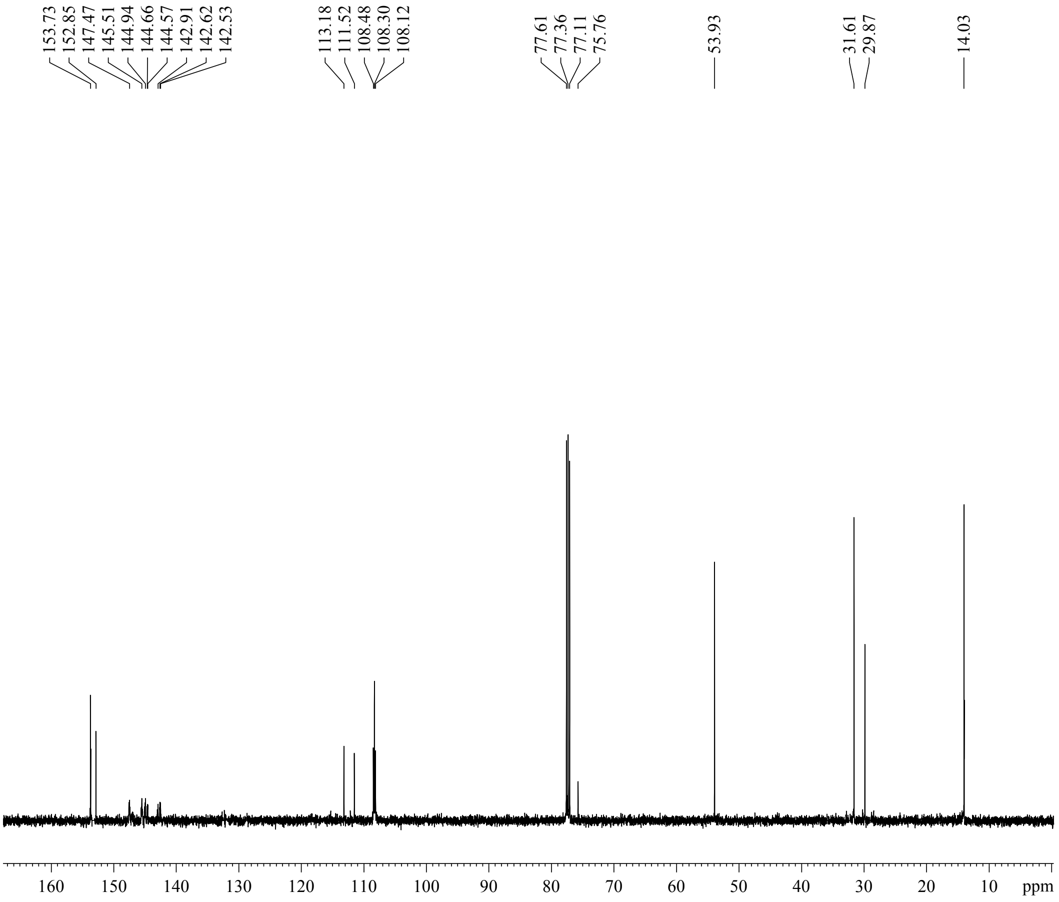

Current Data Parameters
NAME ZX-20140612-LXR-S4
EXPNO 11
PROCNO 1

F2 - Acquisition Parameters
Date_ 20140612
Time 10.20
INSTRUM spect
PROBHD 5 mm PABBO BB-
PULPROG zgpg30
TD 65536
SOLVENT CDCl3
NS 120
DS 4
SWH 29761.904 Hz
FIDRES 0.454131 Hz
AQ 1.1010548 sec
RG 203
DW 16.800 usec
DE 6.50 usec
TE 300.7 K
D1 0.60000002 sec
D11 0.03000000 sec
TD0 1

======== CHANNEL f1 ========
NUC1 13C
P1 9.50 usec
PL1 -1.00 dB
PL1W -1.#IND0000 W
SFO1 125.7703643 MHz

======== CHANNEL f2 ========
CPDPRG2 waltz16
NUC2 1H
PCPD2 80.00 usec
PL2 0 dB
PL12 16.05 dB
PL13 17.00 dB
PL2W -1.#IND0000 W
PL12W -1.#IND0000 W
PL13W -1.#IND0000 W
SFO2 500.1320005 MHz

F2 - Processing parameters
SI 32768
SF 125.7577659 MHz
WDW EM
SSB 0
LB 1.00 Hz
GB 0
PC 1.40

Compound **30** ^{13}C-NMR
